# Optogenetic Reporters Delivered as mRNA Facilitate Repeatable Action Potential and Calcium Handling Assessment in Human iPSC-Derived Cardiomyocytes

**DOI:** 10.1093/stmcls/sxac029

**Published:** 2022-04-16

**Authors:** Loukia Yiangou, Albert Blanch-Asensio, Tessa de Korte, Duncan C Miller, Berend J van Meer, Mervyn P H Mol, Lettine van den Brink, Karina O Brandão, Christine L Mummery, Richard P Davis

**Affiliations:** Department of Anatomy and Embryology, Leiden University Medical Center, Leiden, The Netherlands; Department of Anatomy and Embryology, Leiden University Medical Center, Leiden, The Netherlands; Department of Anatomy and Embryology, Leiden University Medical Center, Leiden, The Netherlands; Department of Anatomy and Embryology, Leiden University Medical Center, Leiden, The Netherlands; Department of Anatomy and Embryology, Leiden University Medical Center, Leiden, The Netherlands; Department of Anatomy and Embryology, Leiden University Medical Center, Leiden, The Netherlands; Department of Anatomy and Embryology, Leiden University Medical Center, Leiden, The Netherlands; Department of Anatomy and Embryology, Leiden University Medical Center, Leiden, The Netherlands; Department of Anatomy and Embryology, Leiden University Medical Center, Leiden, The Netherlands; Department of Applied Stem Cell Technologies, University of Twente, Enschede, The Netherlands; Department of Anatomy and Embryology, Leiden University Medical Center, Leiden, The Netherlands

**Keywords:** human pluripotent stem cells, mRNA, GEVI, GECI, optical analysis, cardiac disease modeling

## Abstract

Electrical activity and intracellular Ca^2+^ transients are key features of cardiomyocytes. They can be measured using organic voltage- and Ca^2+^-sensitive dyes but their photostability and phototoxicity mean they are unsuitable for long-term measurements. Here, we investigated whether genetically encoded voltage and Ca^2+^ indicators (GEVIs and GECIs) delivered as modified mRNA (modRNA) into human induced pluripotent stem cell-derived cardiomyocytes (hiPSC-CMs) would be accurate alternatives allowing measurements over long periods. These indicators were detected in hiPSC-CMs for up to 7 days after transfection and did not affect responses to proarrhythmic compounds. Furthermore, using the GEVI ASAP2f we observed action potential prolongation in long QT syndrome models, while the GECI jRCaMP1b facilitated the repeated evaluation of Ca^2+^ handling responses for various tyrosine kinase inhibitors. This study demonstrated that modRNAs encoding optogenetic constructs report cardiac physiology in hiPSC-CMs without toxicity or the need for stable integration, illustrating their value as alternatives to organic dyes or other gene delivery methods for expressing transgenes.

## Graphical Abstract

**Graphical Abstract:**
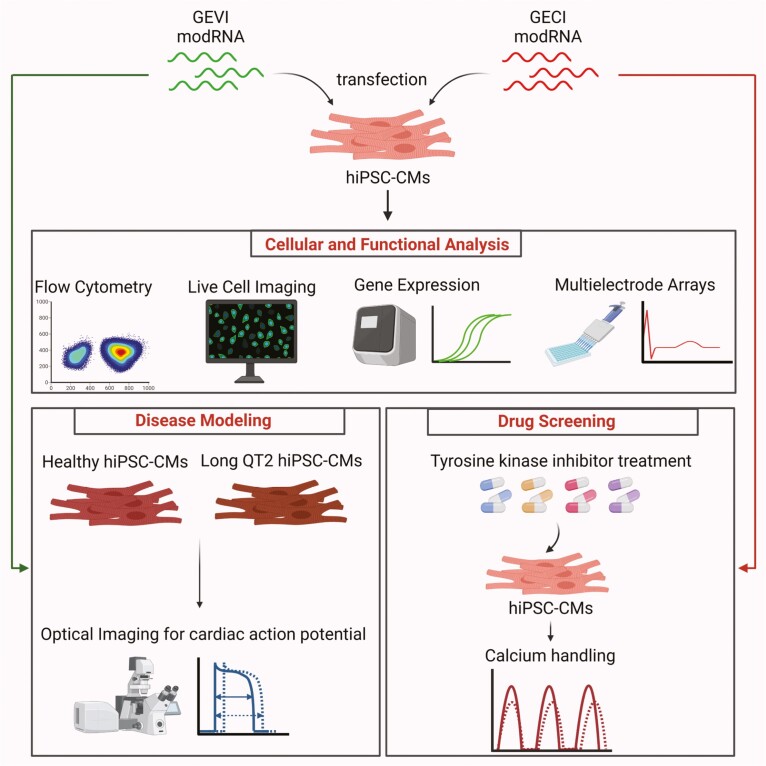

**Significance Statement**

The electrical activity and calcium handling properties of cardiomyocytes are frequently dysregulated in cardiac disease or in response to drugs. Both parameters can be evaluated optically in hiPSC-CMs using organic dyes or optogenetic reporters but photostability issues and toxicity limit flexibility and measurement “on-demand.” This study demonstrates that mRNA encoding optogenetic reporters can be transfected into healthy or diseased hiPSC-CMs to measure their action potentials and calcium transients repeatedly over extended periods. Thus, mRNA-encoded optogenetic reporters are versatile tools able to reveal cardiac disease phenotypes and drug responses rapidly, readily, and repeatedly.

## Introduction

For more than a decade, human-induced pluripotent stem cell-derived cardiomyocytes (hiPSC-CMs) have provided an invaluable tool to study the genetic- and cellular basis of cardiac disease, perform drug screenings, and predict cardiac toxicity.^1-5^ Crucial to this success has been the parallel development of efficient and cost-effective procedures to study cardiac physiology in culture.^6^ Patch-clamp electrophysiology to measure action potential (AP) characteristics remains the gold standard, but this technique requires skilled users, specialist equipment, and is labor intensive and invasive.^7^ This precludes high throughput studies and repeated measurements on the same cells. Multielectrode array (MEA) technology to measure extracellular field potential (FP) characteristics addresses these issues to some extent^8^ and are often used in disease modeling with hiPSC-CMs and for small-scale drug screenings; however, they too require specialist equipment.

Cytosolic Ca^2+^ transients are also important features of cardiac physiology, and changes can affect both AP and contractility kinetics in hiPSC-CMs.^9^ Optical approaches, which in principle address issues of throughput, are the primary methods for measuring Ca^2+^ transients and can be done effectively using small-molecule dyes that detect intracellular Ca^2+^, potentially in combination with voltage-sensitive dyes.^10,11^ However, both voltage- and Ca^2+^-sensitive dyes (VSDs and CSDs) have certain limitations. First, organic dyes can be phototoxic to the cells, which may impact their value in pharmacological safety assessments. Moreover, they may be sensitive to photo-bleaching, diffuse relatively rapidly from the cell membrane, or accumulate in intracellular vesicles.^12,13^ This means cells can generally only be recorded within a short timeframe. Finally, CSDs can also aggregate within different cellular compartments, bind to proteins, and sequester Ca^2+^.^14^

The development and application of genetically encoded voltage indicators (GEVIs) or genetically encoded Ca^2+^ indicators (GECIs) for cardiomyocyte assessment in the early 2000s provided an alternative approach.^15^ Nevertheless, their use was limited in practice due to difficulties in achieving the sensitivity and speed of synthetic indicators. However, further advances have resulted in GEVIs and GECIs with greater sensitivity and improved kinetics, meaning that optogenetic sensors are now a feasible alternative to organic dyes. These indicators are generally composed of a sensing element (ie, voltage-sensing domain for GEVIs; calmodulin domain for GECIs) fused to a fluorescent protein. Upon changes in membrane potential or Ca^2+^ concentration, the sensing element undergoes a conformational change resulting in changes in fluorescence intensity.^15^

In recent years, GEVIs and GECIs have been applied to assess hiPSC-CMs.^16-22^ However, these studies all relied on genomic integration of the optogenetic sensors, either by lentiviral transduction or gene targeting. While lentiviral delivery results in high expression, when performed in human pluripotent stem cells (hPSCs), silencing of the indicators can occur during the differentiation process.^18,19^ For introduction directly into hiPSC-CMs, high multiplicity of infections (MOIs) can be required for efficient transduction,^23^ which may lead to complications with copy number variation and cytotoxicity. Additionally, the procedure leads to the reporters randomly integrating into the genome, which might affect the neighboring genes and their expression. Alternatively, GEVIs or GECIs have been integrated into the AAVS1 safe-harbor locus in hiPSCs.^16,21,24^ While this can support the stable and long-term expression of the reporters in hiPSC-CMs, studies are restricted to the specific hiPSC line targeted. Comparing drug responses or investigating mechanisms in different patient-derived lines, therefore, requires generating transgenic derivatives in each case.

mRNA transfection is rapidly emerging as an attractive alternative to DNA-based systems for delivering genes into cells or tissues. Although expression of genes delivered as in vitro transcribed mRNA previously was hampered by innate immune responses and degradation by RNases,^25^ incorporating modified nucleosides such as pseudouridine and 5-methylcytidine overcomes these limitations and provides robust protein expression.^26^ It has also been demonstrated that modified mRNA (modRNA) can provide robust and transient expression of genes both in cardiomyocytes in vitro and in various animal model hearts in vivo,^27-30^ thereby offering a versatile and fast gene delivery system.

Here, we hypothesized that this system would solve the most acute challenges in the use of GEVIs and GECIs. Thus, we investigated the potential of delivering GEVIs or GECIs as modRNA into hiPSC-CMs to monitor the functional activity of these cells. We found that the GEVI ASAP2f^31^ and the GECI jRCaMP1b^32^ exhibit robust expression with low cytotoxic effects. We demonstrated that this method permits in hiPSC-CMs both the detection of a disease phenotype of Long QT Syndrome, as well as acute and long-term assessment of tyrosine kinase inhibitors (TKIs) known to be associated with a risk of cardiovascular complications.

Overall, we found that modRNA-based delivery of GEVIs and GECIs offers similar flexibility and throughput as that of VSDs and CSDs without phototoxicity and lack of photostability. They thereby provide a robust optical tool for cardiac disease modeling and drug screening studies that can support repeated measurements on the same cardiomyocytes over extended periods.

## Materials and Methods

An extended Methods section is provided in the Supplementary Information.

### Culture of hiPSCs and Differentiation to Cardiomyocytes

The hiPSC lines were maintained, differentiated into cardiomyocytes, and cryopreserved as described in the Supplementary Information. All analyses were performed on cryopreserved hiPSC-CMs 5-10 days after thawing.

### Thawing and Replating of hiPSC-CMs

hiPSC-CMs were thawed and maintained in Medium C (Ncardia) or a modified BPEL medium (mBEL) as previously described.^33,34^ Thawed hiPSC-CMs were replated on Matrigel-coated cell culture plates in medium C or mBEL supplemented with RevitaCell (1:100 dilution; Thermo-Fisher) at a density of 1.6-1.9 × 10^5^ cells/cm^2^ for 96-well plates or 1.3-1.6 × 10^5^ cells/cm^2^ for all other formats. The medium was refreshed 24 h later, and subsequently every 2-3 days thereafter.

### modRNA Synthesis and Transfection

Target sequences were PCR amplified with PrimeSTAR Max DNA polymerase (Takara) using primers carrying the T7 polymerase promoter sequence for in vitro transcription. Following purification, the PCR products were digested with specific restriction enzymes to generate a 5ʹ overhang at the 3ʹ end of template. The digested PCR product was gel extracted using the Wizard SV Gel and PCR Clean-Up System (Promega), concentrated by ethanol precipitation, and resuspended in RNAse-free water (final concentration ~160 ng/µL). The DNA template was transcribed using the INCOGNITO T7 ARCA 5mC- & Ψ-RNA Transcription Kit (Cellscript) following the manufacturer’s instructions. The resulting modRNA was precipitated using LiCl following standard procedures^35^ prior to 5ʹ capping and poly(A) tailing of the purified modRNA using the ScriptCap Cap 1 Capping System and A-Plus Poly(A) Polymerase Tailing Kit respectively (both Cellscript). The resulting capped and tailed modRNA was again precipitated using LiCl, resuspended in RNAse-free water, and quantified. Integrity of the modRNA was confirmed by gel electrophoresis using the Reliant RNA Precast Gel System (Lonza). The target sequences and primers used for PCR amplification are provided in Supplementary Table S1.

The hiPSC-CMs were transfected with modRNAs using Lipofectamine Stem Transfection Reagent (Invitrogen) according to the manufacturer’s instructions. Briefly, 80 ng of modRNA per 5-6 × 10^4^ cells was combined with Opti-MEM (Gibco) and Lipofectamine Stem Transfection Reagent, and incubated for 10 minutes at ~18 °C before being added to the cells. 18-20 h after transfection, the cells were refreshed with Medium C or mBEL.

### Flow Cytometry and Immunofluorescence

Flow cytometric and immunofluorescence analysis were performed as described in the Supplementary Information.

### Apoptosis Analysis

Apoptosis was assessed using Annexin V-PE (Miltenyi Biotec) as described in the Supplementary Information.

### Live Cell Imaging

An EVOS M7000 Imaging System (Thermofisher) equipped with GFP and Texas Red light cubes was used for live imaging of ASAP2f-transfected and jRCaMP1b- or FlicR1-transfected hiPSC-CMs, respectively.

### Gene Expression Analysis

RNA extraction, cDNA synthesis, and RT-qPCR were performed as described in the Supplementary Information.

### Multi-Electrode Array (MEA) Analysis

MEA experiments were performed as described in the Supplementary Information.

### Optical Recordings of AP and Ca^2+^ Transients

AP and Ca^2+^ transient measurements were performed as described in the Supplementary Information.

### Ca^2+^ Transient Assays

Ca^2+^ transient assays and drug screening using the Functional Drug Screening System (FDSS/µCell, Hamamatsu Photonics K.K.) were performed as described in the Supplementary Information.

### Statistical Analysis

Results are presented as mean ± SEM, with a comparison between groups performed using the tests indicated in the figure legend. *P* values <.05 were considered statistically significant. Statistical analyses were performed with GraphPad Prism 8 software.

## Results

### hiPSC-CMs Are Efficiently Transfected with modRNAs

**Figure 1.**
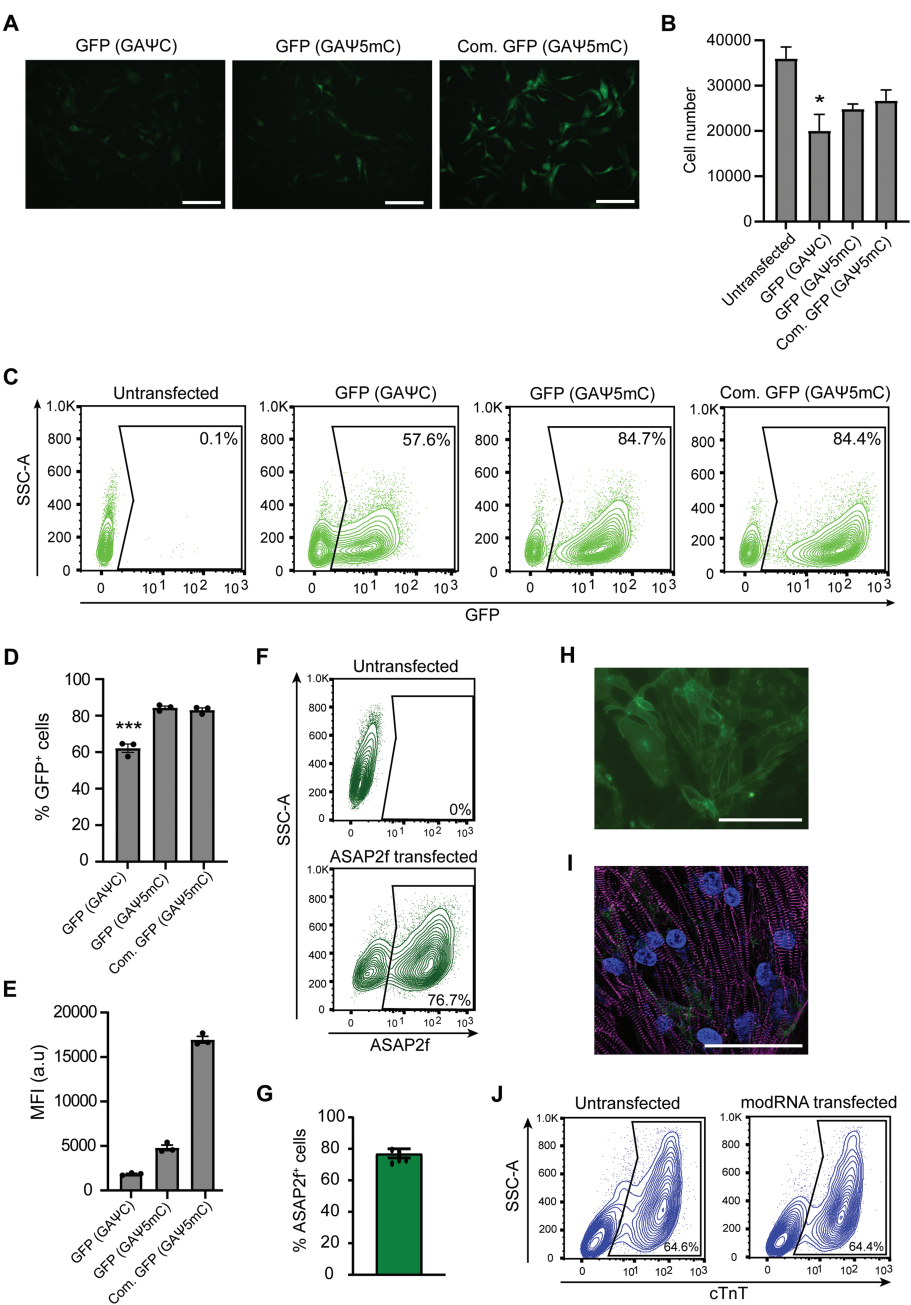
hiPSC-CMs are efficiently transfected with modRNAs. **(A)** GFP expression after transfecting hiPSC-CMs with GFP mRNA containing different modified nucleosides or from different sources. Scale: 100 µm. **(B)** Relative number of hiPSC-CMs recovered following transfection with different GFP modRNA constructs. Data are mean ± SEM (*n* = 3 independent transfections). One-way ANOVA followed by Dunnett’s test for multiple comparisons was performed (*P < .05). **(C)** Representative flow cytometry plots of hiPSC-CMs transfected with GFP mRNA containing different modified nucleosides or from different sources. Values within gated regions indicate % of GFP^+^ cells. **(D-E)** Bar graphs summarizing % of GFP^+^ cells (**D**) and MFI (**E**) after transfection of various GFP modRNA constructs, determined by flow cytometry. Data are mean ± SEM (*n* = 3 independent transfections). One-way ANOVA followed by Dunnett’s test for multiple comparisons was performed (***P < .001). **(F)** Representative flow cytometry plots of hiPSC-CMs transfected with ASAP2f modRNA. Values within the gated regions indicate % of ASAP2f^+^ cells. **(G)** Bar graph summarizing % of ASAP2f^+^ hiPSC-CMs following transfection with ASAP2f modRNA. Data are mean ± SEM (*n* = 6 independent transfections). **(H)** Live cell imaging showing membrane localization of ASAP2f in hiPSC-CMs. Scale = 100 µm. **(I)** Immunofluorescence image of α-actinin (magenta) and ASAP2f (green) expression in transfected hiPSC-CMs. Nuclei (blue) were stained with DAPI. The fluorescence signal for ASAP2f (green) is weak due to the fixation procedure. Scale: 50µm. **(J)** Flow cytometry plots quantifying % of hiPSC-CMs (cTnT^+^) in cultures either untransfected or transfected with ASAP2f modRNA. Values within the gated regions indicate % of cTnT^+^ cells.

To assess whether mRNA could be delivered into hiPSC-CMs, we undertook cellular and molecular analysis of the transfected cells. We first evaluated whether incorporating the modified ribonucleoside bases, 5-methylcytosine-5ʹ-triphosphate (5mCTP) and pseudouridine-5ʹ-triphosphate (ΨTP), into the synthesized mRNA improved cell viability and protein expression. We synthesized GFP-encoding transcripts incorporating either ΨTP only or together with 5mCTP, and also compared these to a commercially available GFP modRNA that included both ΨTP and 5mCTP. More GFP^+^ hiPSC-CMs were observed following the inclusion of both modified nucleosides than ΨTP only, while the total number of hiPSC-CMs recovered following transfection with single modified (GAΨC) mRNA were significantly lower, suggesting that GAΨC mRNA was more cytotoxic (Fig. 1A, 1B; and Supplementary Fig. S1A). Indeed, transfection with GAΨC mRNA resulted in a higher percentage of apoptotic (Annexin V^+^) cells (Supplementary Fig. S1B, S1C). Flow cytometric analysis also confirmed that on average significantly more GFP^+^ cells were obtained from transfecting GFP mRNA containing both modifications (84 ± 1.0% vs. 62 ± 2.3%), as well as an improvement in mean fluorescence intensity (MFI) (4799 ± 308.2 a.u. vs. 1857 ± 75.4 a.u.) (Fig. 1C-1E), indicating higher protein expression. Transfection of a commercially available GFP GAΨ5mC RNA resulted in a similar cell recovery and percentage of hiPSC-CMs that were GFP^+^, validating the improved efficiency observed with double-modified GFP mRNA (Fig. 1B-1D). Notably, the commercial GFP modRNA resulted in significantly brighter GFP^+^ hiPSC-CMs (Fig. 1A, 1E). This could be due to either differences in the GFP sequence or the presence of a longer polyA tail (Supplementary Fig. S1D), thereby improving its stability and expression in the cells. Incorporating both ΨTP and 5mCTP into mRNA encoding the GEVI ASAP2f also improved the percentage of hiPSC-CMs transfected and expression levels (Supplementary Fig. S1E). Taken together, these data indicate the ease and robustness of modRNA transfection as a method for exogenous gene expression in hiPSC-CMs, as well as the advantage of incorporating both ΨTP and 5mCTP ribonucleosides and a polyA tail in the modRNA for improved viability and expression.

We next determined the optimal amount of ASAP2f modRNA to transfect. We found with 75 ng of modRNA that >80% of hiPSC-CMs were transfected, and higher amounts did not improve this further (Fig. 1F-1G; Supplementary Fig. S1F). We also observed that ASAP2f was localized to the cell membrane as expected (Fig. 1H). Furthermore, modRNA transfection did not appear to have detrimental effects on the hiPSC-CM morphology or structural integrity based on the sarcomeric organization visualized by the marker α-actinin (Fig. 1I). Moreover, flow cytometric analysis for the cardiac marker cardiac troponin T (cTnT) indicated the number and percentage of hiPSC-CMs were similar in both untransfected and transfected conditions (Fig. 1J). Finally, transient changes in ASAP2f fluorescence were observed in hiPSC-CMs, with the fluorescence intensity decreasing during the depolarization phase followed by an increase during membrane repolarization (Supplementary Video S1).

We also evaluated the structurally different GEVI, FlicR1.^36^ Contrary to the results obtained with ASAP2f, transfecting hiPSC-CMs with modRNA encoding FlicR1 did not result in functional expression of the protein. Small red fluorescent clusters were seen within the cells with no labelling at the cell membrane (Supplementary Fig. S1G). Flow cytometric analysis also confirmed that fewer hiPSC-CMs (<34%) were FlicR1^+^ (Supplementary Fig. S1H, S1I). For this reason, we focused on GAΨ5mC mRNA-encoding ASAP2f in subsequent experiments.

### ASAP2f modRNA Delivers Strong and Stable Signals in hiPSC-CMs

**Figure 2.**
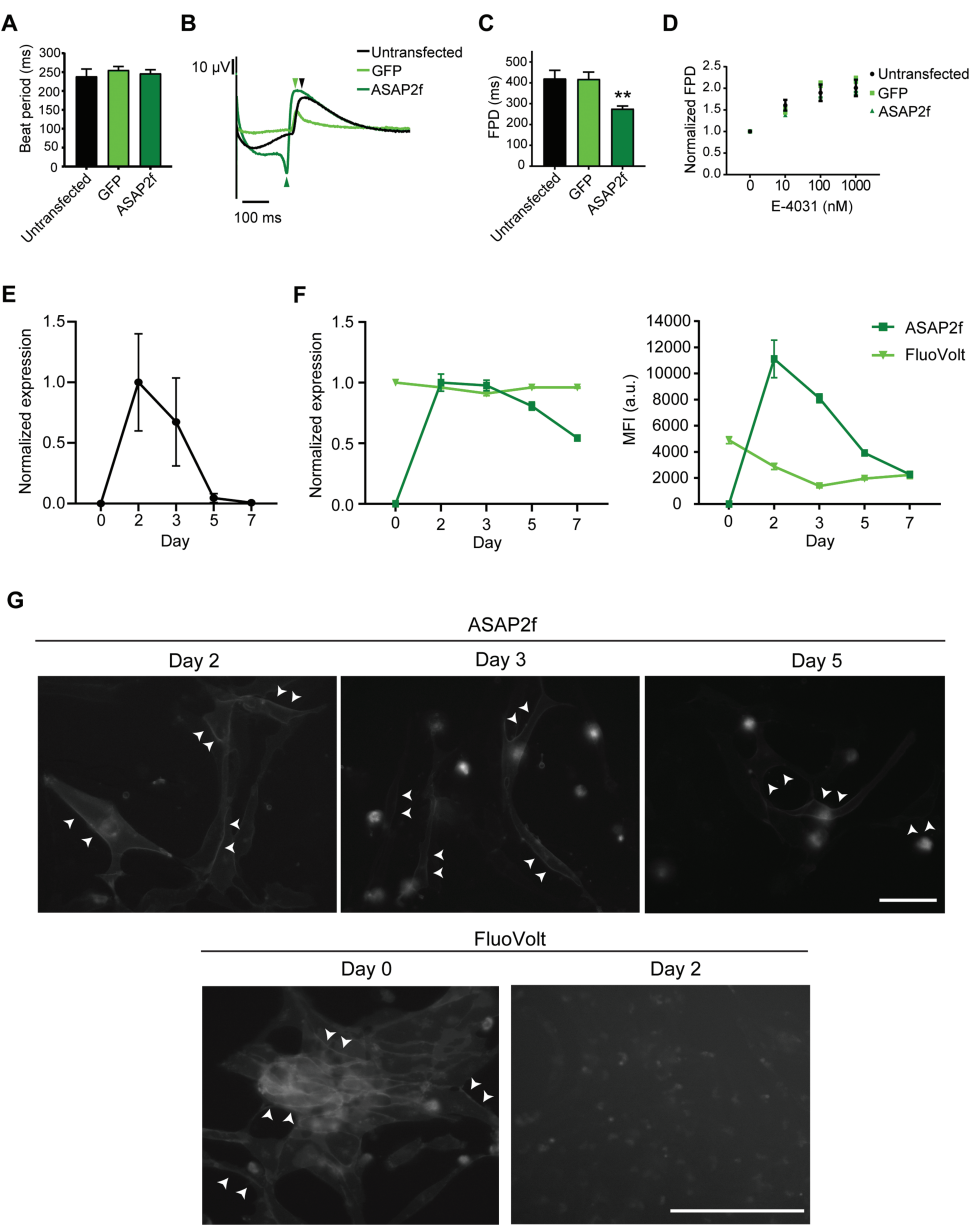
ASAP2f modRNA is strongly expressed in hiPSC-CMs and does not affect their response to E-4031. **(A)** Effect of modRNA transfection on beat period. Data are mean ± SEM (*n* = 8-16 wells from 2 to 4 independent transfections). One-way ANOVA test followed by Dunnett’s test for multiple comparisons was performed. **(B)** Representative field potential (FP) traces following transfection with ASAP2f or GFP modRNA. Arrowheads indicate the repolarization peak for each trace. **(C)** Effect of modRNA transfection on FPD. Data are mean ± SEM (*n* = 13-16 from 2 to 4 independent transfections). One-way ANOVA followed by Dunnett’s test for multiple comparisons was performed (**P < .01 vs. untransfected control). **(D)** Graph of FPD normalized to baseline for indicated transfection conditions upon cumulative addition of E-4031. Data are mean ± SEM (*n* = 13-16 wells from 2 to 4 independent transfections). Two-way ANOVA followed by Tukey’s test for multiple comparisons was performed. **(E)** RT-qPCR analysis for *ASAP2f* expression over a 7-day period following transfection. Data is normalized to the housekeeping gene *RPL37A*, and shown as means ± SEM (*n* = 6 independent transfections). **(F)** Graphs summarizing changes in the relative number of cells that were ASAP2f^+^ or FluoVolt^+^ and their corresponding MFI. Data are mean ± SEM (*n* = 3 independent transfections). **(G)** Live-cell imaging showing expression and localization of ASAP2f and FluoVolt signals at the timepoint when it is strongest, followed by the signal 1-3 days later. Arrowheads indicate examples of membrane labeling. Scale: 100 μm.

To determine whether the expression of ASAP2f altered the function of hiPSC-CMs, we recorded the extracellular FP using MEA technology. We also transfected hiPSC-CMs with GFP modRNA to assess whether modRNA alone affected the electrical activity of the hiPSC-CMs. The beat period was not altered by either ASAP2f or GFP mRNA transfections (Fig. 2A). Notably, transfection of modRNA encoding ASAP2f but not GFP reduced the field potential duration (FPD) compared to untransfected hiPSC-CMs (Fig. 2B, 2C). We, therefore, evaluated whether this shortening caused by the GEVI affected how hiPSC-CMs responded to the AP-prolonging compound, E-4031. With increasing concentrations of E-4031, the FPD was prolonged as expected. This was similar in untransfected, GFP- and ASAP2f-transfected hiPSC-CMs when normalized to baseline values (Fig. 2D), therefore indicating that transfection with modRNA did not affect the response of the hiPSC-CMs to the compound. A similar response was also seen upon correction of FPD (cFPD) using Bazett’s formula to account for the shorter beat period seen in the ASAP2f-transfected hiPSC-CMs at the highest concentration of E-4031 (Supplementary Fig. S2A, S2B).

We further characterized the stability of the ASAP2f signal in the hiPSC-CMs. RT-qPCR analysis over a period of 7 days confirmed the highest expression of the transcript 2 days after transfection, followed by a gradual decrease and loss of the transcript by day 7 (Fig. 2E). Similar kinetics were observed at the protein level, with flow cytometric analysis indicating that both the maximum number of cells expressing ASAP2f and the highest MFI were at day 2 (Fig. 2F). Despite autofluorescence from cell debris and the gradual decrease in MFI over time (Supplementary Fig. S2C, S2D), ASAP2f expression was maintained at a level amenable for imaging up to 5 days after transfection (Fig. 2G, Supplementary Fig. S2D). We compared the signal of ASAP2f to the commonly used voltage-sensitive dye, FluoVolt. Although flow cytometric analysis indicated that the FluoVolt signal was retained by the hiPSC-CMs for at least 7 days (Fig. 2F and Supplementary Fig. S2E), within 2 days the FluoVolt signal had diffused and was no longer clearly present on the membrane (Fig. 2G). Overall, these data indicate that ASAP2f modRNA facilitates greater stability regarding the timeframe in which the hiPSC-CMs can be imaged compared to VSDs.

### ASAP2f modRNA Reports Prolonged APs in a LQT2 hiPSC-CM Model

**Figure 3.**
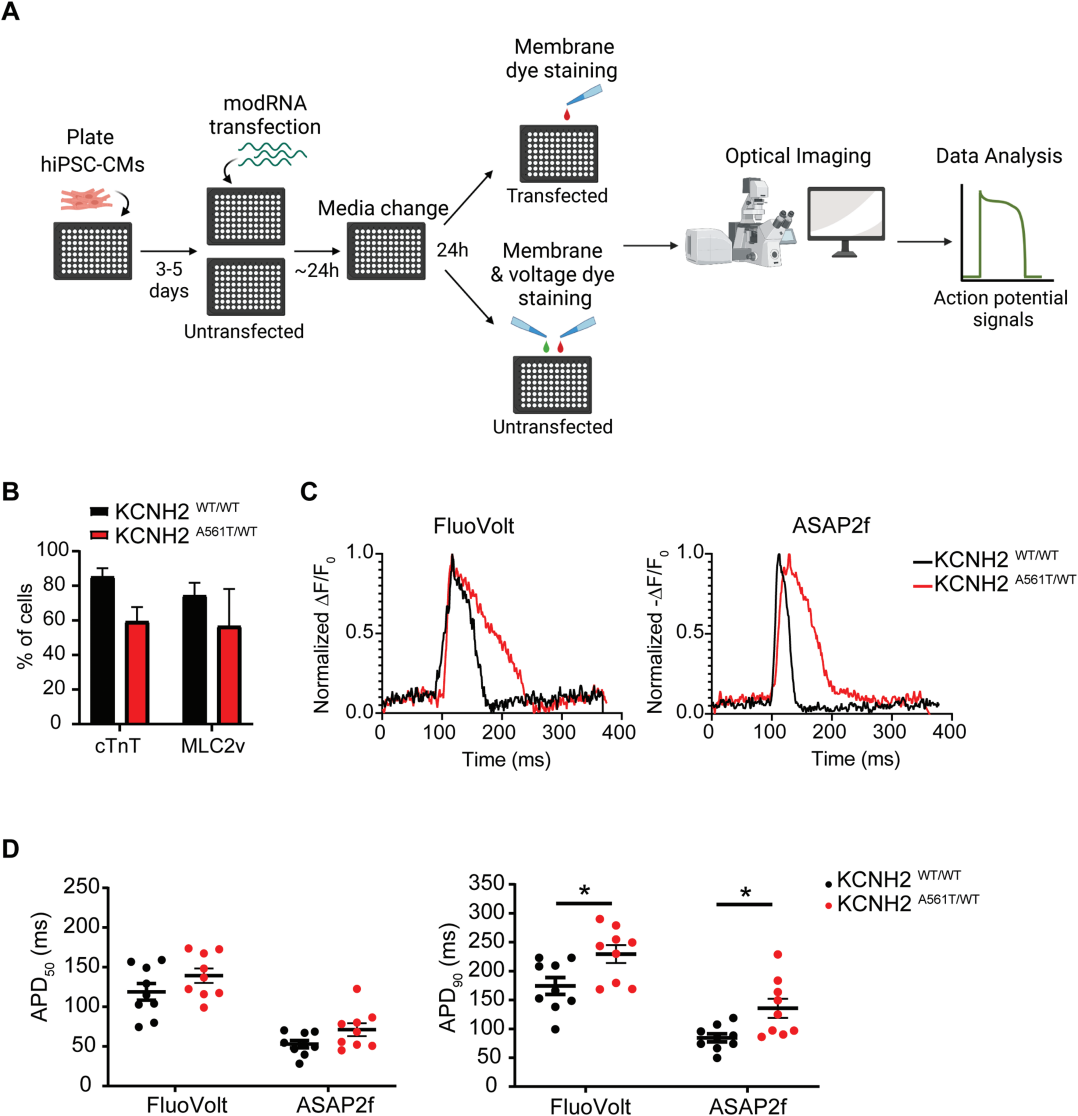
ASAP2f modRNA reports the electrophysiological phenotype in LQT2 hiPSC-CMs. **(A)** Schematic overview of the experimental plan. **(B)** Graph summarizing the percentage of hiPSC-CMs (cTnT) and the proportion of ventricular-like (MLC2v) cardiomyocytes within the hiPSC-CM population for the wildtype (KCNH2^WT/WT^) and LQT2 (KCNH2^A561T/WT^) lines, as determined by flow cytometry. Data are mean ± SEM (*n* = 3 independent differentiations per line). An unpaired *t*-test was performed. **(C-D)** Representative averaged AP transients (C), and average APD_50_ and APD_90_ values (**D**), for wildtype (KCNH2^WT/WT^) and LQT2 (KCNH2^A561T/WT^) hiPSC-CMs as determined following either FluoVolt staining or ASAP2f modRNA transfection. Data are mean ± SEM (*n* = 9 from 3 independent differentiations). Two-way ANOVA followed by Sidak’s test for multiple comparisons was performed (*P < .05).

We next investigated whether we could use modRNA encoding ASAP2f to detect electrophysiological phenotypes in hiPSC models of arrhythmogenic diseases. For this, we examined a pair of isogenic hiPSC lines in which a long QT syndrome type 2 (LQT2)-causing heterozygous missense mutation (KCNH2 p.Ala561Thr) had been introduced into a control hiPSC line.^37^ The hiPSC-CMs from both mutant and wild-type cell lines (KCNH2^A561T/WT^ and KCNH2^WT/WT^, respectively) were either transfected with ASAP2f modRNA or labelled with FluoVolt, before determining their electrophysiological properties (Fig. 3A). When differentiated, the percentage of ventricular-like hiPSC-CMs, as determined by co-expression of cardiac troponin T (cTnT) and myosin light chain 2v (MLC2v) expression, was similar between KCNH2^WT/WT^ hiPSC-CMs (85% ± 5% cTnT^+^ of which 74% ± 7% were MLC2v^+^) compared to the isogenic KCNH2 ^A561T/WT^ hiPSC-CMs (60% ± 8% cTnT^+^ of which 57% ± 21% were MLC2v^+^) (Fig. 3B). Optical recordings confirmed that both reporters could reveal prolonged AP duration (APD) in the KCNH2^A561T/WT^ hiPSC-CMs. As expected, the overall optical APD_90_ values were shorter in hiPSC-CMs transfected with ASAP2f modRNA, but there was still a significant prolongation in the KCNH2 ^A561T/WT^ hiPSC-CMs (136 ± 16.5 ms) compared to the isogenic KCNH2^WT/WT^ hiPSC-CMs (84 ± 7.1 ms) (Fig. 3C, 3D). Also, the overall fold increase in APD_90_ was similar for both fluorescent reporters (FluoVolt: 1.32-fold; ASAP2f: 1.61-fold), as well as that measured previously with the voltage indicator, ANNINE-6plus (1.36-fold).^37^ In summary, these data show that modRNA transfection of ASAP2f offers a viable alternative to VSDs for reporting APs in hiPSC-CMs.

### Measuring Ca^2+^ Transients in hiPSC-CMs with jRCaMP1b modRNA

**Figure 4.**
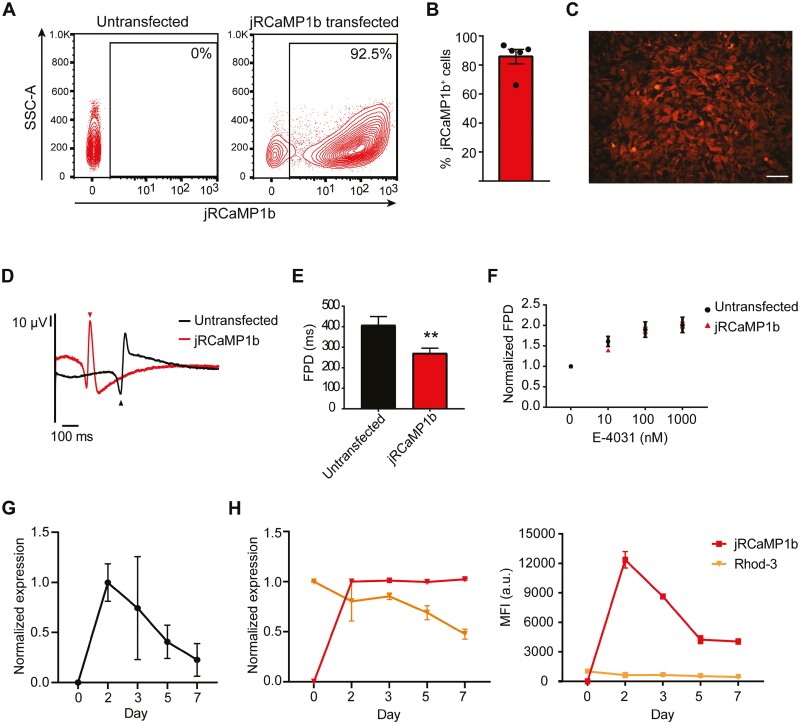
jRCaMP1b modRNA is strongly expressed in hiPSC-CMs and does not affect their response to E-4031. **(A-B)** Representative flow cytometry plots (**A**) and average % (**B**) of jRCaMP1b^+^ cells after transfection. Data are mean ± SEM (*n* = 5 independent transfections). **(C)** Live-cell imaging showing jRCaMP1b expression and cellular localization. Scale: 200 µm. **(D)** Representative FP traces following transfection with jRCaMP1b modRNA. Arrowheads indicate the repolarization peak for each trace. **(E)** Effect of jRCaMP1b modRNA transfection on FPD. Data are mean ± SEM (*n* = 4-16 wells from 1 to 4 independent transfections). Unpaired *t*-test was performed (**P < .01). **(F)** Graph of FPD normalized to baseline for indicated transfection conditions upon cumulative addition of E-4031. Data are mean ± SEM (*n* = 4-16 wells from 1 to 4 independent transfections). Two-way ANOVA followed by Sidak’s test for multiple comparisons was performed. **(G)** RT-qPCR analysis for *jRCaMP1b* expression over a 7-day period following transfection. Data is normalized to the housekeeping gene *RLP37A*, and shown as means ± SEM (*n* = 6 independent transfections). **(H)** Graphs summarizing changes in the relative number of cells that were jRCaMP1b^+^ or Rhod-3^+^ and their corresponding MFI. Data are mean ± SEM (*n* = 3 independent transfections).

Ca^2+^ handling is a key readout in cardiosafety studies.^38^ We, therefore, investigated whether modRNA encoding the GECI jRCaMP1b could be used to monitor cytosolic free-Ca^2+^ transients in hiPSC-CMs. Again we observed consistently high transfection efficiencies, with the majority of hiPSC-CMs (85.6 ± 4.9%) expressing jRCaMP1b (Fig. 4A-C). Furthermore, jRCaMP1b fluorescence cyclically fluctuated with changes in intracellular Ca^2+^ concentrations in the cells, showing an increase with rising intracellular Ca^2+^ levels and a decrease during diastole (Supplementary Video S2).

We further investigated the effect of jRCaMP1b on hiPSC-CM function by measuring the extracellular FPs of the transfected hiPSC-CMs. As with ASAP2f, the beat period was not altered by the transfection (Supplementary Fig. S3A), but jRCaMP1b expression resulted in a shorter FPD compared to untransfected hiPSC-CMs (Fig. 4D, 4E). We additionally assessed the sensitivity of the transfected hiPSC-CMs to E-4031. No differences in the beat period were observed with increasing concentrations of E-4031 (Supplementary Fig. S3B), and both untransfected and jRCaMP1b-transfected hiPSC-CMs showed similar increases in FPD or cFPD over baseline (Fig 4F; Supplementary Fig. S3C).

We subsequently evaluated the stability of the jRCaMP1b signal and compared it to the organic Ca^2+^ indicator Rhod-3, which has similar excitation and emission spectra. RT-qPCR analysis over a 7-day period following transfection showed the jRCaMP1b transcript was most present 2 days after transfection and still detectable on day 7 (Fig. 4G). This was also reflected at the protein level, with jRCaMP1b still measurable on day 7 in all cells that expressed it on day 2 (Fig. 4H; Supplementary Fig. S3D, S3E). In contrast, by day 7 approximately 50% of the cells originally labelled with Rhod-3 were still positive (Fig. 4H; Supplementary Fig.S3F). Additionally, the maximum MFI with Rhod-3 was >12-fold less than the maximum jRCaMP1b signal (Fig. 4H). It was also weak and heterogeneous both intra and intercellularly, in contrast to the bright and uniform expression of jRCaMP1b (Supplementary Fig. S3E, S3G). In sum, these data indicate that jRCaMP1b modRNA provides a brighter, more efficient, and stable reporter to monitor Ca^2+^ handling dynamics compared to CSDs.

### jRCaMP1b modRNA Enables Long-term Monitoring of TKI Effects in hiPSC-CMs

**Figure 5.**
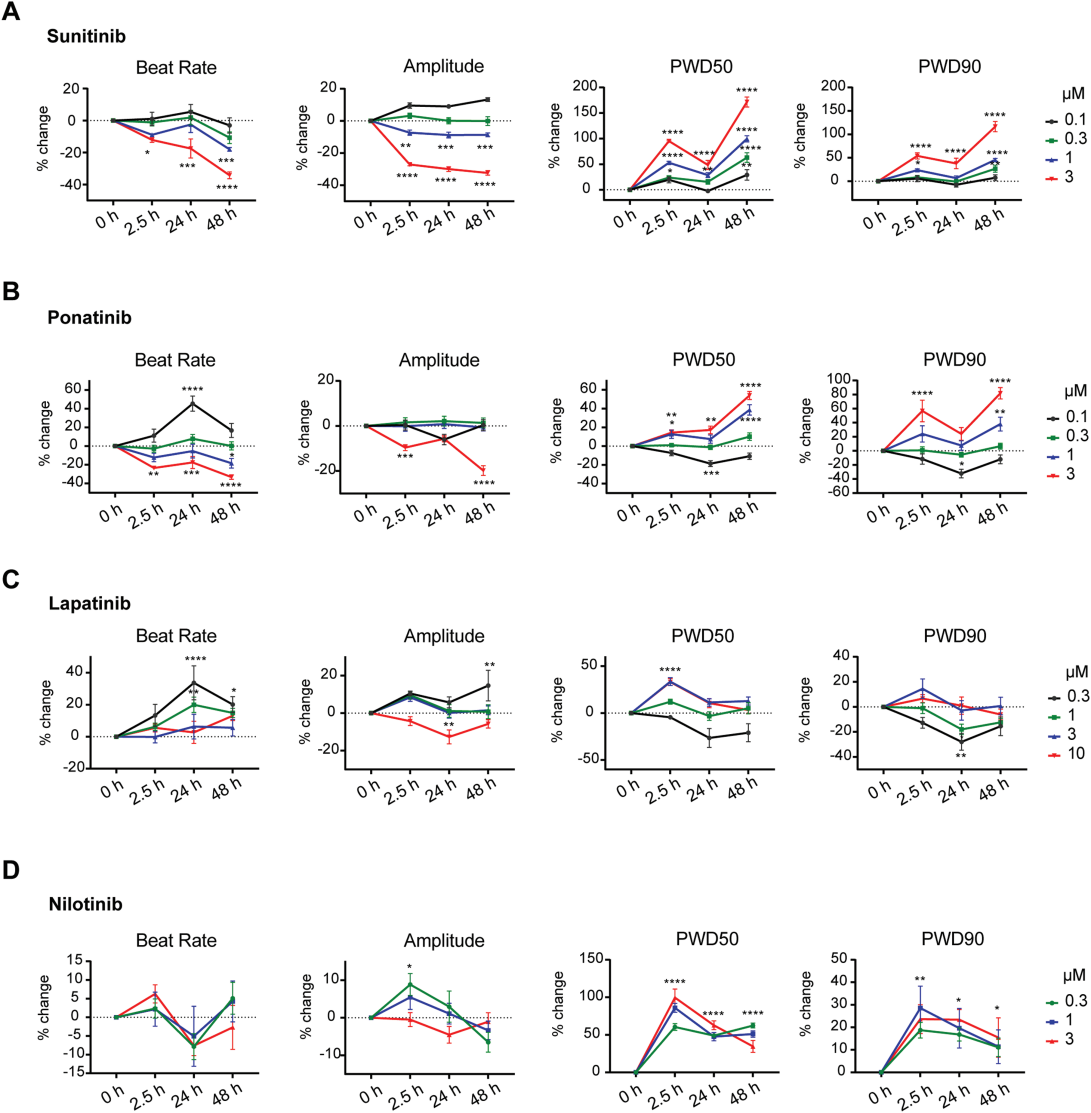
jRCaMP1b modRNA facilitates repeated measurements in TKI-treated hiPSC-CMs. (**A-D**) Relative changes in beat rate, Ca^2+^ peak amplitude, and signal duration represented as Ca^2+^ peak width duration (PWD) at 50% and 90% repolarization over 48 h for hiPSC-CMs treated with varying concentrations of sunitinib (**A**), ponatinib (**B**), lapatinib (**C**) and nilotinib. (**D**) Data are mean ± SEM (*n* = 4-6 wells). Two-way ANOVA followed by Tukey’s test for multiple comparisons was performed (*P < .05, **P < .01, ***P < .001, ****P < .0001).

Next, we investigated whether jRCaMP1b transfected hiPSC-CMs could detect drug-induced changes in Ca^2+^ handling. Specifically, we wished to determine whether introducing the GECI as modRNA enabled the hiPSC-CMs to be repeatedly measured to evaluate both acute and long-term effects on Ca^2+^ transients. We, therefore, assessed beat rate, peak Ca^2+^ amplitude and Ca^2+^ width duration at 50% and 90% decay (PWD_50_ and PWD_90_, respectively) in the same hiPSC-CMs at multiple timepoints over 48 h following the addition of TKIs known to cause cardiotoxicity (Fig. 5).

Sunitinib caused a concentration- and time-dependent reduction in beat rate and systolic Ca^2+^ amplitude, with significant reductions at 1µM and 3µM (Fig. 5A; Supplementary Fig. S4A). There was also a concentration- and time-dependent prolongation in PWD_50_ and PWD_90_, indicating that repeated measurements using jRCaMP1b were able to capture both the acute and long-term effects of sunitinib on Ca^2+^ handling dynamics at clinically relevant concentrations. Similar responses were also observed in hiPSC-CMs treated with 1 µM or 3 µM ponatinib, with both a reduced beat rate and Ca^2+^ peak amplitude, and a prolonged PWD_50_ and PWD_90_ (Fig. 5B; Supplementary Fig. S4B). Again, these changes were greatest 48 h after treatment. Intriguingly, we observed an increased beat rate and shortened PWD at the lowest concentration (0.1 µM) at 24 h, suggesting that at different concentrations this compound could have opposing effects.

For lapatinib, 0.3-1 µM increased the beat rate, peaking at 24 h before starting to decrease (Fig. 5C; Supplementary Fig. S4C). Similarly, 10 µM lapatinib resulted in a transient decrease of the systolic Ca^2+^ amplitude at 24 h. At 3 µM and 10 µM, lapatinib induced an acute PWD_50_ prolongation at 2.5 h, while 0.3 µM lapatinib resulted in a time-dependent shortening of PWD_50_ and PWD_90_. These effects appeared reversible, as the values were not significantly different to baseline measurements at 48 h. Finally, for nilotinib, the peak changes in amplitude and PWD occurred 2.5 h after TKI addition, with the increase in systolic Ca^2+^ amplitude at 0.3µM being transient (Fig. 5D; Supplementary Fig. S4D). Prolongation of PWD_50_ and PWD_90_ was detected over the entire 48 h, albeit decreasing with time.

In summary, these data demonstrate that jRCaMP1b enables repeated measurements of Ca^2+^ handling parameters in hiPSC-CMs at baseline and upon drug treatment, delivering important advantages to CSDs for the purpose of drug screening studies.

### hiPSC-CMs Can Be Co-transfected with Both ASAP2f and jRCaMP1b modRNAs

**Figure 6.**
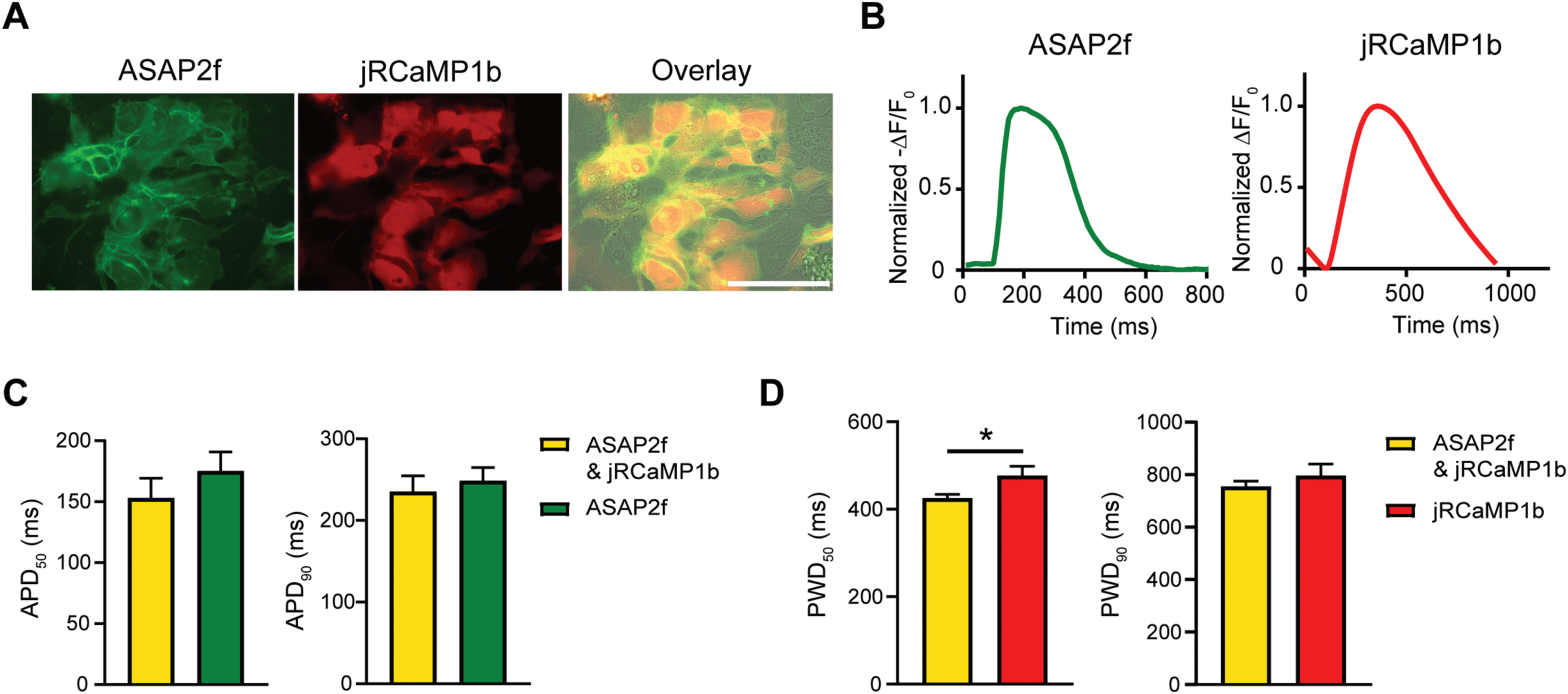
hiPSC-CMs co-transfected with ASAP2f and jRCaMP1b modRNAs report both AP and Ca^2+^ transients. **(A)** Live cell imaging showing co-expression of both ASAP2f and jRCaMP1b in hiPSC-CMs. Scale: 100 µm. **(B)** Representative averaged AP (left) and Ca^2+^ trace (right) in hiPSC-CMs co-transfected with ASAP2f and jRCaMP1b modRNAs. **(C)** Average APD_50_ and APD_90_ values for hiPSC-CMs transfected with ASAP2f only or both ASAP2f and jRCaMP1b modRNAs. Data are mean ± SEM (*n* = 4 independent transfections). Unpaired *t*-test was performed. **(D)** Average PWD_50_ and PWD_90_ values for hiPSC-CMs transfected with jRCaMP1b only or both ASAP2f and jRCaMP1b modRNAs. Data are mean ± SEM (*n* = 4 independent transfections). Unpaired *t*-test was performed (*P < .05).

Lastly, we examined whether the hiPSC-CMs could be co-transfected with both ASAP2f and jRCaMP1b modRNAs, thus facilitating the evaluation of electrophysiological and Ca^2+^ handling properties in the same cardiomyocytes. The hiPSC-CMs expressed both reporters with ASAP2f localized to the cell membrane and jRCaMP1b localized to the cytoplasm (Fig. 6A). Optical measurements for both AP and Ca^2+^ transients could be recorded (Fig. 6B). Furthermore, no significant differences in APD_50_, APD_90,_ or PWD_90_ were observed in co-transfected hiPSC-CMs compared to those transfected only with ASAP2f or jRCaMP1b respectively (Fig. 6C, 6D). Overall, these results confirm that hiPSC-CMs can be simultaneously transfected with 2 reporters, allowing more than one functional parameter to be measured in the hiPSC-CMs.

## Discussion

The generation of tools to study hiPSC-CM physiology that are amenable to scale up are critical to fully utilize this model in understanding cardiac disease mechanisms and performing drug screening. Optical-based technologies are most suited for high throughput use and are extensively used to assess intracellular Ca^2+^ handling and electrical activity in hiPSC-CMs, mainly by organic dyes and genetically encoded reporters. Moreover, these methods are non-invasive and genetic reporters in particular offer the possibility to measure the same cells multiple times.

Several reports have demonstrated the potential of GEVIs and GECIs for electrophysiological and Ca^2+^ handling studies in hPSC-CMs,^15^ although the reporters tested are limited and in-depth comparisons with organic dyes are lacking. In the case of GEVIs, ArcLight and VSFP2.3 are the most widely used,^16,18,19,22^ but both have slower kinetics than organic dyes.^15^ The GEVI, Accelerated Sensor of Action Potentials 1 (ASAP1), improved this by fusing the voltage-sensing domain of a phosphatase from *Gallus gallus* to a circularly permuted GFP,^39^ and was shown to have faster kinetics in reporting APs in cardiomyocytes compared to Arclight.^40^ Further mutagenesis to the linker sequence resulted in ASAP2f,^31^ but no studies to date have compared ASAP2f to other GEVIs or VSDs in hiPSC-CMs. For GECIs, both the GECO (R-GECO1, K-GECO1) and GCaMP (GCaMP5G, GCaMP6f) family of sensors have been used in hPSC-CMs,^17,18,20,41,42^ despite their susceptibility to photoactivation or photoconversion.^13^ Additionally, GFP-based GECIs cannot be combined with channelrhodopsin-2 for the optical pacing of the cells. The mRuby-based GECI, jR-CaMP1b, was developed for use with ChR2 actuation and with improved kinetics over its parental GECI,^32^ although again its use in hPSC-CMs has not been explored.

Here, we advanced the application of genetically encoded indicators in hiPSC-CMs by testing these newer GEVI and GECI iterations, and developing an alternative approach for their cellular delivery. Specifically, when the reporters were in vitro transcribed as modRNA, transfection of ASAP2f and jRCaMP1b was efficient, stable, and less cytotoxic when compared to single nucleoside modified mRNA. Furthermore, these constructs enabled easy optical reporting of either APs or intracellular Ca^2+^ transients in healthy or diseased hiPSC-CMs, that could be repeated in the same cells over several days. Within our experimental setup, the signals were of sufficient quality to measure up to 5 days after transfection. It is important to note that the absolute number of days that sufficient signal is observed depends on the brightness and stability of the reporter, and can vary between different proteins.

It is known that mRNA delivery into human cells can result in an immunogenic response and reduction in mRNA translation that can be mitigated by certain modifications to the mRNA.^43,44^ Comparison of single modified (GAΨC) to double-modified (GAΨ5mC) mRNA in hiPSC-CMs confirmed that GAΨ5mC modRNA resulted in less cell death, as well as increased transfection efficiency and expression of the construct.

In vitro transcription followed by the transfection of modRNA directly into the hiPSC-CMs likewise enables different GEVI and GECI reporters to be rapidly screened. We also investigated whether FlicR1, a GEVI reported to have faster kinetics,^36^ could be used to determine the AP in hiPSC-CMs. However, we observed low levels of expression in hiPSC-CMs and no cell membrane localization, suggesting that this reporter was either not efficiently expressed or trafficked by the cells. Certain modifications may be necessary to achieve efficient expression of the protein in the hiPSC-CMs. For example, altering the length of the linker domain for the GEVI Ace2N-mNeon improved expression in cultured neurons.^45^ Future studies to determine whether other cell types or multicellular constructs also can be transfected in vitro with modRNA, and whether the encoded reporter is expressed and functional will be beneficial. This could include assessing Ca^2+^ dynamics in smooth muscle and endothelial cells, recording neuronal APs, or measuring more mature cardiomyocytes in engineered heart tissues or cardiac microtissues.^46-48^

A key advantage of using GECI-encoding modRNAs over organic dyes is the ability to perform repeated recordings of the hiPSC-CMs. This allowed us to monitor the development of TKI-induced intracellular Ca^2+^ changes in the same hiPSC-CMs over 48 h. Several TKIs commonly used to treat cancers can also cause irreversible cardiac injury in patients, either in the short- or long-term.^49^ It has been suggested that early indications of long-term clinical cardiotoxicity can be detected after short treatment periods in hiPSC-CMs,^4^ with altered intracellular Ca^2+^ handling and impaired Ca^2+^ homeostasis proposed as potential early biomarkers of cardiac damage.^50^ Here, we studied the time- and concentration-dependent effects of the TKIs sunitinib, ponatinib, lapatinib, and nilotinib, on Ca^2+^ handling at clinically relevant concentrations. The jRCaMP1b signal remained stable over the period, enabling assessment of acute, delayed, and transient effects in beat rate, Ca^2+^ peak amplitude, and Ca^2+^ PWD.

For sunitinib and ponatinib the effects on the beat rate and Ca^2+^ dynamics developed over the 48 h period in a concentration- and time-dependent manner. Previous studies have shown similar concentration- or time-dependent effects on beat rate upon treating hiPSC-CMs with these compounds but have not assessed Ca^2+^ handling effects specifically.^51,52^ For lapatinib, higher concentrations resulted in an acute and temporary prolongation of the Ca^2+^ PWD, with the opposite effect developing later at lower concentrations. Finally, for nilotinib, an acute effect on Ca^2+^ PWD was observed at all concentrations at 2.5 h that then became less pronounced at later timepoints, although still significantly prolonged compared to pre-treatment recordings. A similar acute PWD_90_ prolongation was previously reported,^53^ but longer-term responses were not evaluated. The degree of change in the Ca^2+^ parameters measured in the hiPSC-CMs may also reflect the varying degrees of cardiotoxicity observed with the TKIs examined. Sunitinib (Sutent) is frequently associated with clinically detectable adverse cardiac effects, including drug-induced cardiomyopathies.^54^ We also observed that this compound produced the largest effects on the Ca^2+^ dynamics in the hiPSC-CMs. Treatment with lapatinib resulted in the smallest effects on Ca^2+^ handling parameters in hiPSC-CMs, which seem to be reversible. These observations are in line with clinical observations where cardiotoxicity associated with lapatinib (Tykerb) is sporadic and less severe in patients, and most often is linked to reversible decreases in left ventricular ejection fraction.^55,56^ As such, combining GECIs to monitor Ca^2+^ handling with viability assays, would provide further insight into the wide range of drug-induced cardiotoxicities that can occur with anti-cancer drugs. In addition, it would be of interest to further utilize the jRCaMP1b modRNA to investigate abnormal Ca^2+^ handling in genetic disease models such as catecholaminergic polymorphic ventricular tachycardia (CPVT), LQT8, or LQT14-16 in which calcium homeostasis is dysregulated.

Despite the benefits of using GEVI and GECI modRNA to study cardiac physiology, they do have certain limitations. While transfection of modRNA enables the same hiPSC-CMs to be repeatedly measured, over time the modRNA is degraded and the signal from the reporter is lost. For assessments beyond 5-10 days, the cells would likely need to be transfected again. Additional studies are needed to determine whether this might further affect the functionality of the hiPSC-CMs. Alternatively, incorporating other modified ribonucleoside bases, such as 1-methylpseudouridine-5ʹ-triphosphate, might help prolong and improve the signal.^29^

We also observed a shortening of the FPD when the hiPSC-CMs were transfected with ASAP2f or jRCaMP1b modRNA. This appears related to the reporters as it was not observed in cells transfected with GFP modRNA. Such analysis has not been rigorously performed in other hiPSC-CM studies that have utilized ArcLight or the GCaMP or GECO sensors, and future studies should determine if this is a general phenomenon with GEVIs and GECIs. It is known that commonly used synthetic voltage and Ca^2+^ dyes can also disrupt cardiomyocyte function.^57,58^ However, the observed FPD shortening did not alter the response of the hiPSC-CMs to the AP-prolonging drug E-4031, nor did it affect detecting a prolonged APD in a LQT2 hiPSC-CM model. Indeed, the relative fold increase in APD_90_ was similar to that obtained with the VSDs, FluoVolt (this study) or ANNINE-6plus.^37^ It will be interesting to study whether there are also effects on ion channel currents or additional cellular processes.

In summary, we demonstrated that transfection of hiPSC-CMs with GEVI or GECI modRNAs can be used for non-invasive evaluation of electrophysiological and intracellular Ca^2+^ handling, with both drug-induced or disease-specific changes faithfully reported. Furthermore, we demonstrated that hiPSC-CMs can be co-transfected with both reporters and their functional parameters recorded within the same cells, allowing for multi-parameter acquisition from a single transfection experiment. Overall, this approach avoids the need to genetically modify hiPSCs and thereby provides a versatile method to functionally characterize hiPSC-CMs differentiated from various hiPSC lines. It also expedites the ability to rapidly assess newly developed variants in the cell type of interest. Moreover, these reporters are stably expressed in the hiPSC-CMs for several days, thereby allowing the cells to be repeatedly monitored which to date is not possible with VSDs or CSDs.

## Supplementary Material

sxac029_suppl_Supplementary_Figure_S1

sxac029_suppl_Supplementary_Figure_S2

sxac029_suppl_Supplementary_Figure_S3

sxac029_suppl_Supplementary_Figure_S4

sxac029_suppl_Supplementary_Material

sxac029_suppl_Supplementary_Table_S1

sxac029_suppl_Supplementary_Table_S2

sxac029_suppl_Supplementary_Video_S1

sxac029_suppl_Supplementary_Video_S2

sxac029_suppl_Supplementary_Legends

## Data Availability

The data underlying this article will be shared on reasonable request to the corresponding author.
