# Supplementary figures and images for: Optogenetic Reporters Delivered as mRNA Facilitate Repeatable Action Potential and Calcium Handling Assessment in Human iPSC-Derived Cardiomyocytes
Source: Stem Cells. 2022 Apr 16;40(7):655–68. doi: 10.1093/stmcls/sxac029 (PMC9332902; doi:10.1093/stmcls/sxac029)

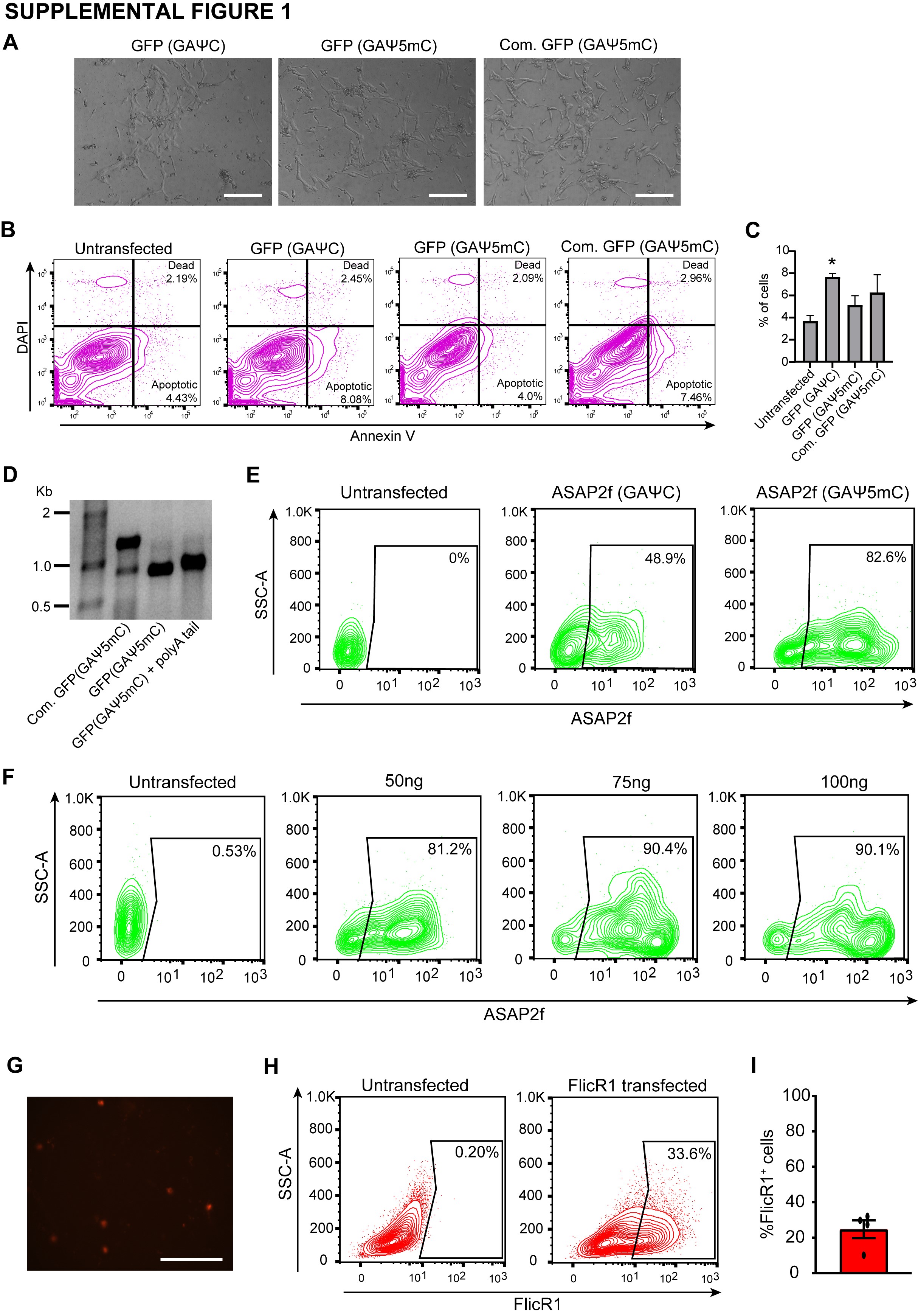

Supplement: sxac029_suppl_Supplementary_Figure_S1 [file sxac029_suppl_supplementary_figure_s1.jpeg]

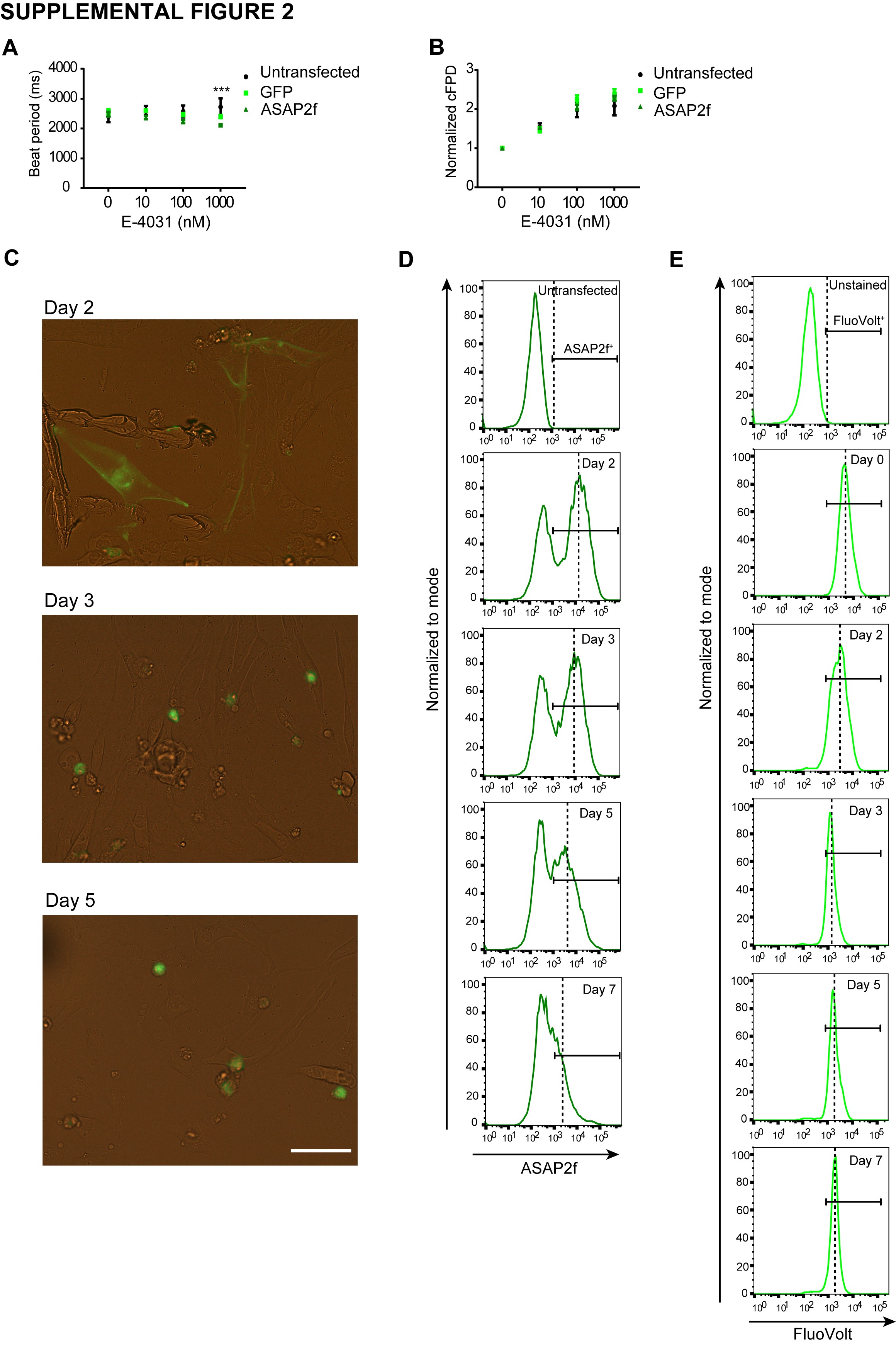

Supplement: sxac029_suppl_Supplementary_Figure_S2 [file sxac029_suppl_supplementary_figure_s2.jpeg]

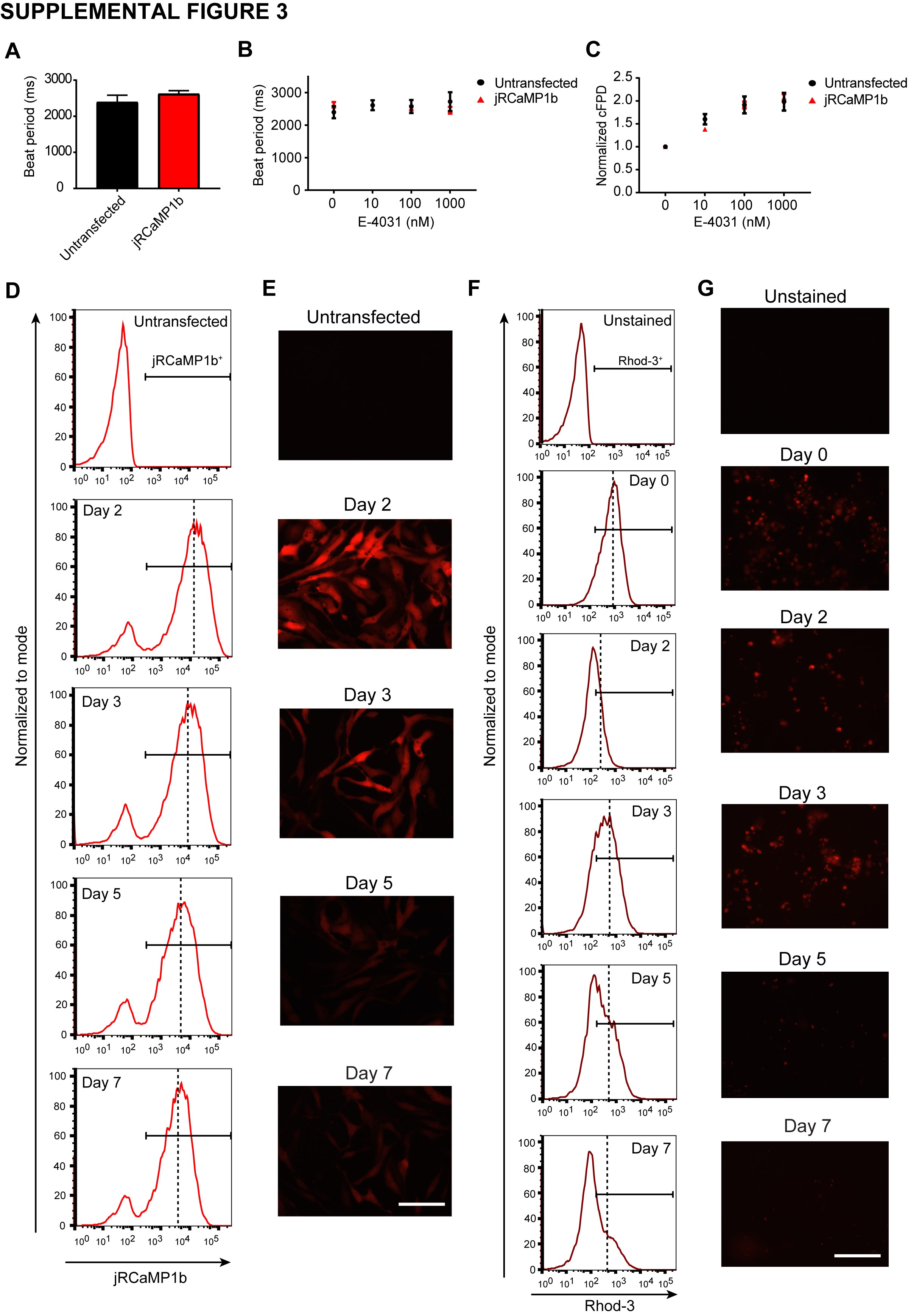

Supplement: sxac029_suppl_Supplementary_Figure_S3 [file sxac029_suppl_supplementary_figure_s3.jpeg]

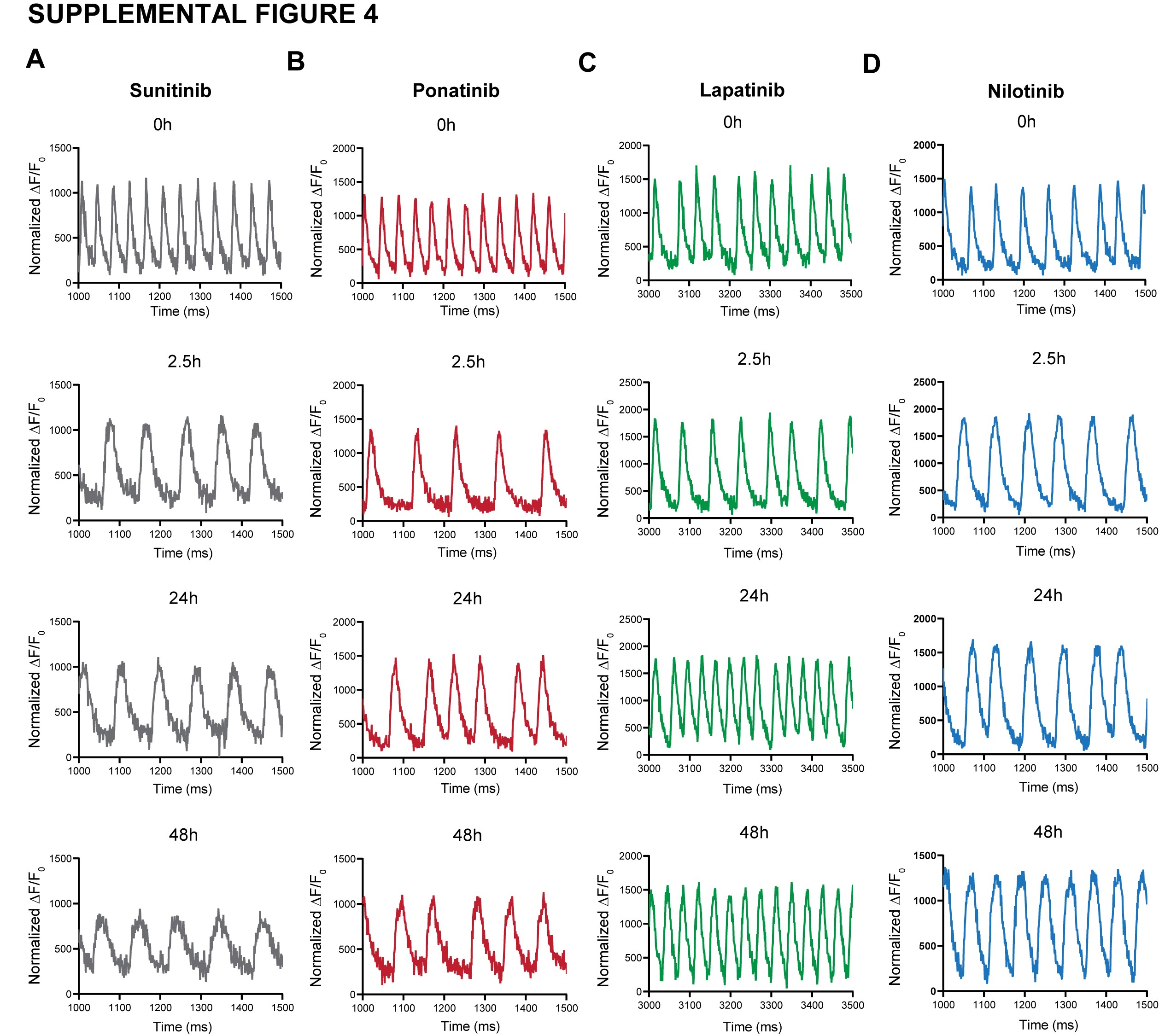

Supplement: sxac029_suppl_Supplementary_Figure_S4 [file sxac029_suppl_supplementary_figure_s4.jpeg]
