# Supplementary material for: Optogenetic Reporters Delivered as mRNA Facilitate Repeatable Action Potential and Calcium Handling Assessment in Human iPSC-Derived Cardiomyocytes
Source: Stem Cells. 2022 Apr 16;40(7):655–68. doi: 10.1093/stmcls/sxac029 (PMC9332902; doi:10.1093/stmcls/sxac029)
Supplement: sxac029_suppl_Supplementary_Material [file sxac029_suppl_supplementary_material.docx]

**SUPPLEMENTAL INFORMATION**

**SUPPLEMENTAL METHODS**

**Culture of hiPSCs and differentiation to cardiomyocytes**

The hiPSC line LUMC0020iCTRL and its derivatives [1,2] were maintained either in Essential 8 or StemFlex Medium (both Gibco). One day prior to differentiation, the hiPSCs were harvested using TrypLE Select (Gibco) and plated onto Matrigel (Corning)-coated wells of a 12-well cell culture plate, either in Essential 8 containing RevitaCell Supplement (1:200 dilution; Gibco) or StemFlex medium. The hiPSCs were differentiated into cardiomyocytes using the Pluricyte Cardiomyocyte Differentiation Kit (Ncardia) according to the manufacturer's instructions. The hiPSC-CMs were maintained in Medium C (Ncardia) until differentiation day 20–21 and then dissociated and cryopreserved as previously described [3].

**Flow cytometry**

A single cell suspension of hiPSC-CMs was obtained by dissociating the cells with TrypLE™ Select Enzyme (Gibco) and filtering the cell suspension. As required, cells were fixed and permeabilized using the Fix & Perm Cell Permeabilization Kit (Invitrogen) according to the manufacturer’s instructions, and incubated with the antibodies, cTnT-Vioblue (1:11, Miltenyi Biotec, #130-106-686) and MLC2v-PE (1:11, Miltenyi Biotec, #130-106-183). Samples were acquired using a MACSQuant VYB flow cytometer (Miltenyi Biotec), and data analyzed using FlowJo software (FlowJo, LLC).

**Apoptosis Analysis**

One day after transfecting the hiPSC-CMs, the medium was collected and kept until hiPSC-CMs were collected for the apoptosis assay. Enumeration of apoptotic and dead cells was determined by flow cytometric analysis using the antibody Annexin V-PE (Miltenyi Biotec, #130-118-363) and DAPI. Briefly, the hiPSC-CMs were dissociated with TrypLE™ Select Enzyme and washed with 1× Annexin V Binding Buffer (Miltenyi Biotec, #130-092-820). The cells and supernatant collected the day after the transfection were pelleted and further resuspended in 98 µl of 1× Annexin V Binding Buffer and mixed with 2 µl of Annexin V-PE for 15 min at RT. Prior to running on a MACSQuant VYB flow cytometer, 0.1 mg/ml of DAPI was added. Data was analyzed using FlowJo software.

**Immunofluorescence analysis**

The hiPSC-CMs plated on glass coverslips were fixed using the Inside Stain Kit (Miltenyi Biotec) according to manufacturer’s instructions. The fixed cells were incubated with α-actinin (1:400, Sigma-Aldrich, #A7811) antibodies followed by Alexa Fluor 594- (1:250, ThermoFisher, #A-21203) conjugated secondary antibody for 1 h at RT. Nuclei were stained with DAPI and images captured using a confocal laser scanning microscope SP8 (Leica) at 63x magnification.

**RNA Extraction, cDNA synthesis and RT-qPCR**

Total RNA was isolated using the Nucleospin RNA kit (Macherey-Nagel) according to the manufacturer’s instructions. Up to 1 μg of RNA was reverse transcribed using the iScript-cDNA Synthesis kit (Bio-Rad). RT-qPCR was performed using iTaq Universal SYBR Green Supermix (Bio-Rad), with samples run on a CFX384 real-time system (Bio-Rad). Results were analyzed using the ΔCt method and gene expression levels normalized to the *RPL37A* housekeeping gene. Primer sequences used for qRT-PCR are provided in Supplemental Table 2.

**Optical recordings of AP and Ca^2+^ transients**

The hiPSC-CMs plated either on black/clear 96-well flat bottom tissue culture-treated microplates (Falcon) or 96-well glass-bottom plates (Greiner) were transfected with modRNA 3-5 days after seeding, and imaged 2-3 days after transfection. For staining with the organic dyes, hiPSC-CMs seeded 5-8 days earlier were incubated with either FluoVolt or Rhod-3 (both 1:833 dilution; ThermoFisher) for 20 min at 37°C. The cells were then washed and left to recover for 10 min at 37°C before analysis. Recordings and data processing were performed using a bespoke fast optical switch microscopy system or a Leica DMI6000 B inverted microscope and algorithms developed in-house, as described previously [4].

**Multi-electrode array (MEA) analysis**

MEA experiments were performed using 96-well plates with gold electrodes (Multichannel Systems). The MEAs were coated with 3 µl human fibronectin (40 µg/ml, Alfa Aesar) for 1 h at 37°C, and hiPSC-CMs seeded directly on the electrodes at a density of 2.5 x 10^4^ cells/well. Medium was changed at least 1 h before baseline recordings. All measurements were made at 37°C in Medium C and at least 15 min after leaving the cells to stabilise in the recording system. Extracellular recordings were performed using a Multiwell-MEA-System (Multichannel systems). For evaluating E-4031-induced effects on the hiPSC-CMs, sequential addition of increasing concentrations of E-4031 was performed. E-4031 (Tocris) was dissolved in dimethylsulfoxide (DMSO; Sigma-Aldrich) at 10 mM, with serial dilutions made in Medium C. The final concentration of the drug was achieved by stepwise removal of medium, and addition of the same volume of diluted E-4031 to the well. No more than 7% of the total volume was replaced. The response to each E-4031 concentration was recorded for 1 min after an incubation period of 1 min. FP traces were analysed using the Multi-Well Analyzer software (Multichannel Systems) to quantify FPD and peak-to-peak intervals over the whole recording time. Bazett’s formula for frequency correction was used to correct for the rate-dependency of FPD (cFPD).

**Calcium transient assays**

The hiPSC-CMs plated on a 96-well black/clear flat bottom tissue culture-treated microplate were transfected with jRCamP1b modRNA 3-5 days after seeding, and the calcium transient assay performed 2-3 days after transfection. The assay was conducted using the Functional Drug Screening System (FDSS/µCell, Hamamatsu Photonics K.K.), equipped with temperature control modules. The compounds used were: Sunitinib malate (1398; Axon Medchem), Lapatinib ditosylate (1395; Axon Medchem), Nilotinib (HY-10159; MedChemExpress LLC) and Ponatinib (S1490; Selleckchem). All compounds were prepared from 10 mM stock solutions in DMSO, diluted in mBEL medium to 10x the final desired concentrations and distributed in a 96-well plate. The assay plate containing the jRCamP1b-expressing hiPSC-CMs was refreshed with medium >2 h before the start of the assay. At least 30 min before recording, the assay plate was placed in the FDSS/µCell system for equilibration. The spontaneous beating activity of jRCaMP1b-expressing hiPSC-CMs was assessed through measuring the calcium fluorescence signal integrated over the whole well. Baseline recordings were performed for 10 min followed by addition of the compound. 10 µl of the 10-fold concentrated compound solution was manually injected from the compound plate (pre-warmed at 37°C) to the wells in the assay plate that already contained 90 µl of cell culture medium (final concentration 0.1% DMSO). jRCaMP1b signals were recorded at: 2.5 h, 24 h and 48 h after compound addition for 5-10 min. The hiPSC-CMs were maintained at 37°C during the acquisition time and the fluorescence signals were recorded at a sampling interval of 60 ms, x255 gain, 2x2 binning and with a LED power of 200 mA. The following parameters were captured and analyzed using the WaveChecker software (Hamamatsu Photonics K.K.): peak rate (beat rate in beats per minute [BPM]), calcium peak amplitude, and calcium peak width duration at 50% and 90% of repolarization (PWD50 and PWD90). All data were normalized to baseline and corrected for the vehicle-control (0.1% DMSO).

**REFERENCES**

1 Zhang M, D’Aniello C, Verkerk AO, et al. Recessive cardiac phenotypes in induced pluripotent stem cell models of Jervell and Lange-Nielsen syndrome: Disease mechanisms and pharmacological rescue. Proc Natl Acad Sci 2014;111:E5383–E5392.

2 Brandão KO, van den Brink L, Miller DC, et al. Isogenic Sets of hiPSC-CMs Harboring Distinct KCNH2 Mutations Differ Functionally and in Susceptibility to Drug-Induced Arrhythmias. Stem Cell Reports 2020;15:1127–1139.

3 van den Brink L, Brandão KO, Grandela C, et al. Cryopreservation of human pluripotent stem cell-derived cardiomyocytes is not detrimental to their molecular and functional properties. Stem Cell Res 2020;43:101698.

4 van Meer BJ, Krotenberg A, Sala L, et al. Simultaneous measurement of excitation-contraction coupling parameters identifies mechanisms underlying contractile responses of hiPSC-derived cardiomyocytes. Nat Commun 2019;10:4325.
