# Supplementary material for: Optogenetic Reporters Delivered as mRNA Facilitate Repeatable Action Potential and Calcium Handling Assessment in Human iPSC-Derived Cardiomyocytes
Source: Stem Cells. 2022 Apr 16;40(7):655–68. doi: 10.1093/stmcls/sxac029 (PMC9332902; doi:10.1093/stmcls/sxac029)
Supplement: sxac029_suppl_Supplementary_Table_S1 [file sxac029_suppl_supplementary_table_s1.docx]

**Supplemental Table 1: Sequences of primers and templates used for IVT**

| **Primer Name** | **Sequence (5’-3’)** |
| --- | --- |
| T7_ASAP2f_FWD | GCAGATGAATTCTAATACGACTCACTATAGGGCATGAGCTAGCCGCCACCATG |
| T7_jRCaMP1b_FWD | GAATTCTAATACGACTCACTATAGGGTAGCCGCCGCCATGGTCGACTCATCGCGACGTAAGTG |
| T7_jRCaMP1b_REV | ATGTGGTATGGCTGATTATGATCTAGAGTCGCGGC |
| T7_FlicR1_FWD | GAATTCTAATACGACTCACTATAGGGAGAGAAGCCACCATGGAGGGATTCGAC |
| T7_REV (used for ASAP2f and FlicR1) | ATGGCTGGCAACTAGAAGGCACAGTCG |
| ASAP2f sequence | GCAGATGAATTCTAATACGACTCACTATAGGGCATGAGCTAGCCGCCACCATGGAGACGACTGTGAGGTATGAACAGGGGTCAGAGCTCACTAAAACTTCGAGCTCTCCAACAGCAGATGAGCCCACGATAAAGATTGATGATGGTCGTGATGAGGGTAATGAACAAGACAGCTGTTCCAATACCATTAGGAGAAAAATTTCCCCGTTTGTGATGTCATTTGGATTCAGAGTATTTGGAGTTGTGCTTATCATTGTAGACATCATAGTGGTGATTGTGGATCTGGCCATCAGTGAGAAGAAAAGAGGCATTAGAGAGATTCTTGAAGGTGTTTCCCTGGCTATAGCACTCTTCTTCCTTGTTGATGTTCTCATGAGAGTGTTTGTTGAAGGCTTCAAGAACTATTTCCGGTCCAAACTGAATACTTTGGATGCAGTCATAGTAGTGGGCACTCTGCTAATTAATATGACCTACTCCTTCTCTGACCTTAGCTTTAACAGCCATAACGTGTATATTACCGCGGATAAACAGAAAAACGGCATTAAAGCGAACTTTACCGTGCGCCATAACGTGGAAGATGGCAGCGTGCAGCTGGCGGATCATTATCAGCAGAACACCCCGATTGGCGATGGCCCGGTGCTGCTGCCGGATAACCATTATCTGAGCACCCAGACCGTGCTGAGCAAAGATCCGAACGAAAAACGCGATCACATGGTGCTGCTGGAATTTGTGACCGCAGCGGGCATTACACACGGCATGGATGAACTGTATGGCGGCACCGGCGGCAGCGCGAGCCAGGGCGAAGAACTGTTTACCGGCGTGGTGCCGATTCTGGTGGAACTGGATGGCGATGTGAACGGCCATAAATTTAGCGTGCGCGGCGAAGGCGAAGGCGATGCGACCATTGGCAAACTGACCCTGAAATTTATTTGCACCACCGGCAAACTACCGGTGCCGTGGCCGACCCTGGTGACCACCTTAACCTATGGCGTGCAGTGCTTTAGCCGCTATCCGGATCATATGAAACGCCATGATTTTTTTAAAAGCGCGATGCCGGAAGGCTATGTGCAGGAACGCACCATTAGCTTTAAAGATGATGGCAAATATAAAACCCGCGCGGTGGTGAAATTTGAAGGCGATACCCTGGTGAACCGCATTGAACTGAAAGGCACCGATTTTAAAGAAGATGGCAACATTCTGGGGCATAAACTGGAATATAACACAGATCAGATGCCGCAGATGGTTACTCTTCTTCGAGTTCTGAGAATTGTTATCTTAATAAGAATATTTCGCCTGGCTTCACAGAAGAAACAACTTGAAGTGGTAACCTAATGAAAGCTTGGTACCGAGCTCGGATCCACTAGTCCAGTGTGGTGGAATTCTGCAGATATCCAGCACAGTGGCGGCCGCTCGAGTCTAGAGGGCCCGTTTAAACCCGCTGATCAGCCTCGACTGTGCCTTCTAGTTGCCAGCCAT |
| jRCaMP1b sequence | GAATTCTAATACGACTCACTATAGGGTAGCCGCCGCCATGGTCGACTCATCGCGACGTAAGTGGAATAAGTGGGGTCACGCAGTCAGAGCTATAGGTCGGCTGAGCTCAGCGAACAACACCGAAATGATGTACCCAGCGGATGGTGGTCTGCGTGGTTACACTCACATGGCGCTGAAAGTTGATGGCGGCGGTCACCTGTCCTGTTCTTTCGTGACCACCTACCGCTCCAAAAAGACTGTCGGCAACATTAAGATGCCTGCCATTCATTACGTCAGCCACCGTCTGGAGCGCCTGGAGGAGAGCGATAACGAAATGTTTGTCGTACAGCGTGAACACGCAGTTGCCAAGTTTGTGGGCCTGGGTGGTGGCGGCGGTACCGGAGGGAGCATGAACTCCCTGATCAAGGAGAACATGCGTATGAAAGTGGTTCTGGAAGGCTCCGTAAACGGCCACCAGTTCAAATGCACTGGTGAAGGCGAAGGCAACCCGTATATGGGCACCCAGACTATGCGTATCAAAGTGATCGAGGGTGGTCCGCTGCCGTTTGCGTTCGACATCCTGGCGACGTCCTTTATGTATGGCTCCCGTACCTTCATCAAATATCCGAAAGGCATCCCGGATTTCTTTAAGCAGTCCTTCCCGGAAGGTTTTACCTGGGAACGTGTGACCCGTTACGAAGACGGCGGCGTAATTACCGTTATGCAAGACACGTCTCTGGAGGATGGCTGCCTGGTGTATCACGTGCAGGTTCGCGGTGTGAACTTCCCGAGCAATGGTGCTGTAATGCAAAAGAAAACCAAAGGTTGGGAGCCTACGGACTCCCAACTGACTGAAGAGCAGATCGCAGAATTTAAAGAGGCTTTCTCCCTATTTGACAAGGACGGGGATGGGACAATAACAACCAAGGAGATGGGGACGGTGATGCGGTCTCTGGGGCAGAACCCCACAGAAGCAGAGCTGCAGGACATGATCAATGAAGTAGATGCCGACGGTGACGGCACAATCGACTTCCCTGAGTTCCTGATTATGATGGCAGGCAAAATGAAATACACAGACAGTGAAGAAGAAATTAGAGAAGCGTTCGGCGTGTTTGATAAGGATGGCAATGGCTACATCAGTGCAGCAGAGCTTCGCCACGTGATGACAAACCTTGGAGAGAAGTTAACAGATGAAGAGGTTGATGAAATGATCAGGGAAGCAGACAGCGATGGGGATGGTCAGGTAAACTACGAAGAGTTTGTACAAATGATGACAGCGAAGTAGGCGGCCGCGACTCTAGATCATAATCAGCCATACCACAT |
| FlicR1 sequence | GAATTCTAATACGACTCACTATAGGGAGAGAAGCCACCATGGAGGGATTCGACGGTTCAGATTTTAGTCCTCCAGCTGATTTAGTTGGCGTTGGCGGTGCAGTCATGCGGAACGTCGTTGACGTCACGATAAATGGTGACGTCACTGCTCCGCCGAAAGCAGCGCCAAGAAAAAGTGAATCGGTAAAGAAAGTTCATTGGAATGATGTAGACCAAGGACCGAGTGAAAAACCAGAGACAAGACAGGAGGAACGAATAGATATACCCGAGATTTCAGGTCTATGGTGGGGCGAGAATGAACATGGAGTGGGCGGTGGGAGAATGGAGATACCTACTACTGGTGTAGGTCGCGTCCAGTTTCGTGTCCGAGCAGTGATTGATCATCTAGGGATGCGAGCCTTTGGAGTCTTCCTAATTCTCTTGGACATCATCCTCATGATCATTGATCTCAGTCTTCCAGGAAAAAGTGAATCTTCACAATCCTTTTATGACGGGTTGGCTTTGGCTCTTTCTTGTTATTTCATGCTGGATTTAGGATTAAGGATATTTGCCTACGGGCCCAAGAATTTCTTCACCAACCCCTGGGAGGTTGCTGATGGCTTGATTATCGTTGTCACATTCGTCGTCACGATATTTTACACTGTGTTAGATGAATACTTTCAAGAAACAGGAGCCGATGGTTTGGGGCAGTTGGTTGTGTTGGCCCGTTTGCTGCGTGTGGTTCGATTAGCAAGAATATTTTATTCCCATCAACAAAGGGTGGTTTCCGAGCGGATGTACCCCGAGGACGGCGTCCTGAAGAGCGAGATCAAGAAGGGGCTGAGGCTGAAGGACGGCGGCCACTACGCCGCCGTGGTCAAGACCACCTACAAGGCCAAGAAGCCCGTGCAGCTGCCCGGCGCCTACATCGTCGACATCAAGTTGGACATCGTGTCCCACAACGAGGACTACACCATCGTGGAACAGTGCGAACGCGCCGAGGGCCGCCACTCCACCGGCGGCATGGACGAGCTGTACAAGGGAGGTACAGGCGGGAGTCTGGTGAGCAAGGGCGAGGAGGTTAACAAGGCCATCATCAAGGAGTTCATGCGCTTCAAGGTGCACATGGAGGGCTCCGTGAACGGCCACGAGTTCGAGATCGAGGGCGAGGGCGAGGGCCGCCCCTACGAGGCCTTTCAGACCGCTAAGCTGAAGGTGACCAAGGGTGGCCCCCTGCCCTTCGCCTGGGACATCCTGTCCCCTCAGTTCATGTACGGCTCCAAGGCCTACATTAAGCACCCAGCCGACATCCCCGACTACTTCAAGCTGTCCTTCCCCGAGGGCTTCAGGTGGGAGCGCGTGATGAACTTCGAGGACGGCGGCATTATTCACGTTAACCAGGACTCCTCCCTGCAGGACGGCGTATTCATCTACAAGGTGAAGCTGCGCGGCACCAACTTCCCCCCCGACGGCCCCGTAATGCAGAAGAAGACCATGGGCTGGGAGGCTACGCGTTAGGGAGGTTGTATGAGTTGCAAATGTGTATTATCCTAGTCTAGAGGGCCCGTTTAAACCCGCTGATCAGCCTCGACTGTGCCTTCTAGTTGCCAGCCAT |
