# Supplementary material for: Optogenetic Reporters Delivered as mRNA Facilitate Repeatable Action Potential and Calcium Handling Assessment in Human iPSC-Derived Cardiomyocytes
Source: Stem Cells. 2022 Apr 16;40(7):655–68. doi: 10.1093/stmcls/sxac029 (PMC9332902; doi:10.1093/stmcls/sxac029)
Supplement: sxac029_suppl_Supplementary_Table_S2 [file sxac029_suppl_supplementary_table_s2.docx]

**Supplemental Table 2: Sequence of Primers used for RT-qPCR**

| **Gene** | **Forward Primer (5’-3’)** | **Reverse Primer (5’-3’)** |
| --- | --- | --- |
| *RPL37A* | GTGGTTCCTGCATGAAGACAGTG | TTCTGATGGCGGACTTTACCG |
| *ASAP2f* | TATGTGCAGGAACGCACCAT | CCATCTGCGGCATCTGATCT |
| *jRCaMP1b* | TCGACTCATCGCGACGTAAG | GAGTGTAACCACGCAGACCA |
