# Supplementary material for: Optogenetic Reporters Delivered as mRNA Facilitate Repeatable Action Potential and Calcium Handling Assessment in Human iPSC-Derived Cardiomyocytes
Source: Stem Cells. 2022 Apr 16;40(7):655–68. doi: 10.1093/stmcls/sxac029 (PMC9332902; doi:10.1093/stmcls/sxac029)
Supplement: sxac029_suppl_Supplementary_Legends [file sxac029_suppl_supplementary_legends.docx]

**Figure S1. hiPSC-CMs are efficiently transfected with modRNAs. Related to Figure 1.**

**(A)** Brightfield images of hiPSC-CMs transfected with GFP mRNA containing different modified nucleosides or from different sources. Scale: 100µm.

**(B)** Representative flow cytometry plots of hiPSC-CMs transfected with different ASAP2f modRNA constructs and labelled with Annexin V and DAPI. Annexin V^+^/DAPI^-^ cells are apoptotic, while Annexin V^+^/DAPI^+^ cells are dead.

**(C)** Graph summarizing flow cytometry results of Annexin V/DAPI staining. Data are mean ± SEM (n=3 independent transfections). One-way ANOVA followed by Dunnett’s test for multiple comparisons was performed (*p<0.05).

**(D)** Denaturing agarose gel electrophoresis showing size (kilobase, Kb) of commercial GFP modRNA in comparison to in-house IVT GFP modRNA before and after polyA tailing.

**(E)** Representative flow cytometry plots of hiPSC-CMs transfected with different ASAP2f modRNA constructs. Values within the gated regions indicate % of ASAP2f^+^ cells.

**(F)** Representative flow cytometry plots of hiPSC-CMs transfected with various amounts of ASAP2f GAΨ5mC modRNA. Values within the gated regions indicate % of ASAP2f^+^ cells.

**(G)** Live cell imaging showing expression and localization of FlicR1 in hiPSC-CMs. Scale: 200µm.

**(H)** Representative flow cytometry plots of hiPSC-CMs transfected with FlicR1 modRNA. Values within the gated regions indicate % of FlicR1^+^ cells.

**(I)** Bar graph summarizing % of FlicR1^+^ hiPSC-CMs following transfection with FlicR1 modRNA. Data are mean ± SEM (n=4 independent transfections).

**Figure S2. ASAP2f modRNA is strongly expressed in hiPSC-CMs and does not affect their response to E-4031. Related to Figure 2.**

**(A)** Graph of beat period for indicated transfection conditions upon cumulative addition of E-4031. Data are mean ± SEM (n=8-16 wells from 2-4 independent transfections). Two-way ANOVA test was performed (***p< 0.001).

**(B)** Graph of corrected FPD (cFPD) normalized to baseline for indicated transfection conditions upon cumulative addition of E-4031. Data are mean ± SEM (n=8-16 wells from 2-4 independent transfections). Two-way ANOVA test was performed.

**(C)** Live cell imaging showing fluorescence and brightfield overlays of ASAP2f transfected hiPSC-CMs. Scale: 100μm.

**(D-E)** Representative flow cytometry plots of hiPSC-CMs transfected with ASAP2f modRNA (D) or stained with FluoVolt (E) during a time-course of 7 days. Dotted lines show MFI.

**Figure S3. jRCaMP1b modRNA is strongly expressed in hiPSC-CMs and does not affect their response to E-4031. Related to Figure 4.**

**(A)** Effect of jRCaMP1b modRNA transfection on beat period. Data are mean ± SEM (n=16 wells from 4 independent transfections). Unpaired t-test was performed.

**(B)** Graph of beat period for indicated transfection conditions upon cumulative addition of E-4031. Data are mean ± SEM (n=16 wells from 4 independent transfections). One-way ANOVA test was performed.

**(C)** Graph of corrected FPD (cFPD) normalized to baseline for indicated transfection conditions upon cumulative addition of E-4031. Data are mean ± SEM (n=16 wells from 4 independent transfections). One-way ANOVA test was performed.

**(D)** Histograms showing percentage of hiPSC-CMs expressing jRCaMP1b over 7 days after transfection. Dotted lines indicate the MFI of the positive population at each time point.

**(E)** Live cell imaging showing expression and localization of jRCaMP1b signals over 7 days after transfection. Scale: 200µm.

**(F)** Histograms showing percentage of hiPSC-CMs labelled with Rhod-3 over 7 days following staining.

**(G)** Live cell imaging showing labelling efficiency and localization of Rhod-3 signals over 7 days after transfection. Scale: 200µm.

**Figure S4.** **jRCaMP1b modRNA facilitates repeated measurements in TKI-treated hiPSC-CMs. Related to Figure 5.**

**(A-D)** Representative Ca^2+^ traces of hiPSC-CMs treated with sunitinib (**A**), ponatinib (**B**), lapatinib (**C**) and nilotinib (**D**) over 48 h.

**Video S1. Video recording of hiPSC-CMs transfected with ASAP2f modRNA.**

**Video S2. Video recording of hiPSC-CMs transfected with jRCaMP1b modRNA.**
